# Specificity, Versatility, and Continual Development: The Power of Optogenetics for Epilepsy Research

**DOI:** 10.3389/fncel.2018.00151

**Published:** 2018-06-14

**Authors:** Zoé Christenson Wick, Esther Krook-Magnuson

**Affiliations:** Graduate Program in Neuroscience and Department of Neuroscience, University of Minnesota, Minneapolis, MN, United States

**Keywords:** seizures, cell type specificity, neuronal circuitry, channelrhodopsin, halorhodopsin, archaerhodopsin, parvalbumin, intersectional genetics

## Abstract

Optogenetics is a powerful and rapidly expanding set of techniques that use genetically encoded light sensitive proteins such as opsins. Through the selective expression of these exogenous light-sensitive proteins, researchers gain the ability to modulate neuronal activity, intracellular signaling pathways, or gene expression with spatial, directional, temporal, and cell-type specificity. Optogenetics provides a versatile toolbox and has significantly advanced a variety of neuroscience fields. In this review, using recent epilepsy research as a focal point, we highlight how the specificity, versatility, and continual development of new optogenetic related tools advances our understanding of neuronal circuits and neurological disorders. We additionally provide a brief overview of some currently available optogenetic tools including for the selective expression of opsins.

## Introduction

**Figure 1:**
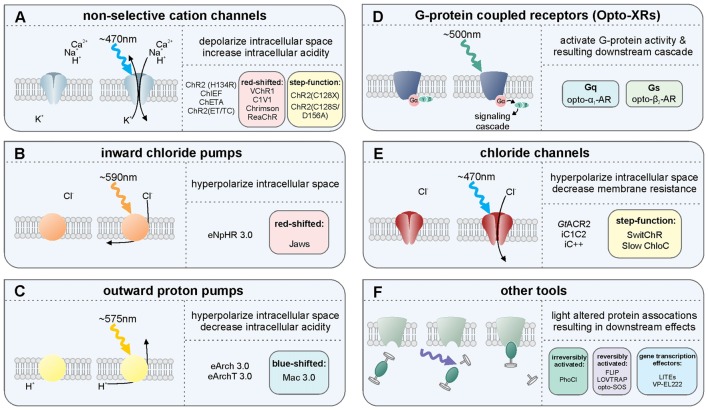
Overview of several light-sensitive proteins, their variants, and their effect on neurons. Throughout, refer to Box 1 for more information on the light-sensitive proteins listed. **(A)** The non-selective cation channels are perhaps the best-known family of excitatory opsins, with several variants stemming from the blue-light activated wild-type channelrhodopsin-2 found in *Chlamydomonas reinhardtii*. These non-selective cation channels allow for passive flow of protons and potassium, sodium, and calcium ions along their electrochemical gradient upon photostimulation with light at approximately 470 nm. In mammalian neurons, opening of these channels results in depolarization of the intracellular space, thus increasing the likelihood of action potential generation. The inward flow of protons has also been shown to affect intracellular pH. Several non-selective cation channel opsin variants are listed including a selection of the many blue-light activated opsin variants: ChR2(H134R), ChR2(C128X), ChiEF, ChETA and ChR2(ET/TC) variants; the red-shifted opsins: VChR1, C1V1, Chrimson and ReaChR; and the step function opsin (SFO) ChR2 (C128X), and stabilized SFO ChR2(C128S/D156A). **(B)** The inward chloride pumps are, to date, perhaps the most commonly used family of inhibitory opsins. Several of these inward chloride pumps, including the one listed (eNpHR3.0) are variants of the wild-type halorhodopsin (HR) found in *Natronomonas pharaonis*. These opsins are activated by 580 nm light and pump one chloride ion into the cell for each photon of light. The red-shifted opsin Jaws is a cruxhalorhodopsin derived from *Haloarcula salinarum* engineered to produce large inhibitory photocurrents induced by red light exposure. These inward chloride pumps hyperpolarize the intracellular space by increasing the intracellular chloride concentration, thus decreasing the likelihood of action potential generation. **(C)** There are several outward proton pumps used in mammalian neuron systems to silence neuronal activity as well. The outward proton pump opsins listed are eArch3.0, eArchT3.0 and eMac3.0, though there are several other versions not listed here. Each has enhanced membrane trafficking and slightly differing photoactivation/deactivation kinetics and resulting photocurrents. Peak photoactivation for eMac3.0 is stimulated by green wavelengths of light and is thus blue-shifted compared to eArch3.0 and eArchT3.0. Each of these outward proton pumps effectively inhibits neuronal activity by hyperpolarizing the intracellular space when protons are pumped out of the cell. Reducing the intracellular concentration of protons also reduces intracellular acidity. **(D)** G-protein coupled receptors indirectly affect neuronal activity by directly activating G proteins and their downstream targets, producing a signaling cascade. Opto-XRs are a family of G-protein coupled receptor opsins including the specific G_q_-protein and G_s_-protein coupled variants. **(E)** Chloride channel opsins allow passive flow of chloride ions down their electrochemical gradient upon photostimulation. In healthy adult mammalian neurons, photoactivation of these chloride channel opsins typically hyperpolarizes the intracellular space, in addition to decreasing membrane resistance, and thus reduce the likelihood of action potential generation. **(F)** There are several other optogenetic systems available that allow for photo-sensitive alteration of intracellular protein activity. Illustrated here is the irreversibly activated photocleavable protein, PhoCl. Other optogenetic protein alteration systems allow reversible alteration of protein-protein interactions, including FLIP, LOVTRAP and opto-SOS. Additional tools allow for light-altered gene expression, including LITEs and VP-EL222.

**Figure 2:**
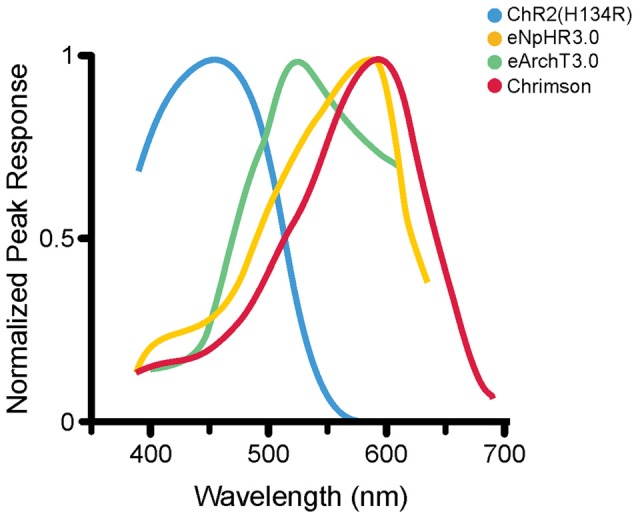
Photoactivation spectra. Action spectra from several opsins; each has a different peak excitation wavelength (shown here) as well as different photocurrent amplitudes and activation and deactivation kinetics (not shown here). ChR2(H134R; Nagel et al., 2005) is maximally excited by blue wavelengths of roughly 450 nm (illustrated activation spectrum for ChR2(H134R) modified from reference; Lin et al., 2009). eNpHR3.0 (Gradinaru et al., 2010) is a commonly used HR variant that is maximally excited at 590 nm (eNpHR3.0 activation spectrum modified from reference; Gradinaru et al., 2010). eArchT3.0, an outward proton pump, is maximally excited at roughly 520 nm (spectrum modified from reference; Mattis et al., 2012). Chrimson is a non-selective cation channel opsin, maximally activated at roughly 590 nm (spectrum modified from reference; Klapoetke et al., 2014). The diverse peak excitation wavelengths make it theoretically possible to do multi-color optogenetic experiments selectively targeting and manipulating different cell populations with reduced cross-talk (Gradinaru et al., 2010; Klapoetke et al., 2014). Note that different experimental conditions can produce different activation spectra; where possible, the illustrated activation spectra are taken from the first manuscript detailing the specific opsin variant illustrated. For an article directly comparing the induced photocurrents of various opsins, including some illustrated here, the reader is referred to reference Mattis et al. (2012).

**Figure 3:**
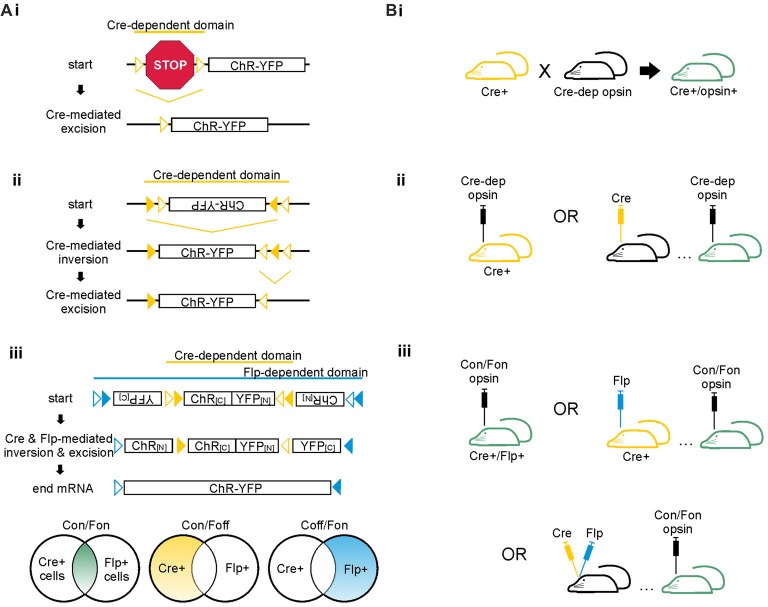
Cre-dependent strategies for achieving cell-type specific opsin expression. **(A)** Schematics of Cre-dependent expression strategies of the excitatory opsin channelrhodopsin (ChR) tagged with yellow fluorescent protein (YFP). (i) Two loxP sites (open triangles) flank a STOP codon that prevents expression of the ChR-YFP construct. Cre can mediate either inversion or excision of DNA located between two loxP sites. If the loxP sites are oriented in the same direction, Cre excises the DNA between the two sites; if the loxP sites are oriented in opposing directions, Cre inverts the DNA between the two sites. In the case shown here, Cre mediates excision of the floxed STOP codon, allowing expression of the opsin. (ii) The FLEX/DIO system utilizes one set of mutant loxP sites (closed triangles) and one set of typical loxP sites (open triangles). Each loxP site will only pair with its match. In this example, Cre mediates inversion of the opsin DNA between the mutant loxP sites. Cre then mediates excision of the mutant between the non-mutant loxP sites, preventing the DNA from flipping back into the anti-sense direction. (iii) The INTRSECT system utilizes both Cre- and Flp-mediated recombination to achieve opsin expression in cell populations defined by two markers. The Cre- and Flp-dependent viral vector illustrated is also known as a Cre-on/Flp-on system, or more simply: Con/Fon. Other options for INTRSECT approaches that allow selective opsin expression are diagramed below, including Cre-on/Flp-off (Con/Foff) and Cre-off/Flp-on (Coff/Fon). Con/Fon vector schematic modified with permission from Fenno et al. (2014). **(B)** Options for achieving cell-type specific expression using the floxed STOP system (i), the FLEX/DIO system (ii), or the INTRSECT system (iii) in mice. Transgenic mice expressing Cre are shown in yellow; mice with ultimate opsin expression through transgenic and/or viral methods are indicated in green. (i) To achieve Cre-dependent opsin expression using the floxed STOP system, one can use a transgenic approach in which a Cre-expressing mouse is crossed with a mouse expressing a Cre-dependent opsin, resulting in offspring that express the opsin Cre-dependently. Viral based methods for Cre expression are also possible, as shown for ii and iii. Viral vectors using the floxed STOP system for opsin expression are also theoretically possible but can result in unwanted/leaky expression of the opsin. Thus, the FLEX/DIO system for Cre-dependent opsin expression was created. (ii) In the FLEX/DIO system, a Cre-dependent opsin vector can be delivered into a transgenic Cre+ mouse or a mouse previously injected with a vector for expression of Cre. (iii) The Con/Fon INTRSECT viruses lead to opsin expression when injected into either Cre+/Flp+ transgenic mice, Cre+ mice injected with a vector encoding Flp-recombinase, Flp+ mice injected with a vector encoding Cre-recombinase (not shown), or mice previously injected with vectors encoding Cre and Flp-recombinases.

Optogenetics rests on the use of genetically encoded light sensitive proteins including opsins (Figure 1, Box 1; Deisseroth, 2011, 2015; Boyden, 2015). Through exogenous expression of these light sensitive proteins, researchers gain the ability to modulate neuronal activity, intracellular signaling pathways, or gene expression through the delivery of light (Figure 2). Spatially and temporally restricted delivery of light provides optogenetic approaches with both temporal and spatial specificity. The use of different light-sensitive proteins provides an additional element of experimental control, including the direction of modulation (e.g., excitation vs. inhibition of neuronal populations). Finally, the restricted expression of light-sensitive proteins allows cell-type specific modulation, that is, selectivity for which cell populations are directly affected when light is delivered (Figure 3, Box 2).

Box 1 **A brief overview of key light-sensitive proteins, including opsins.**

**The non-selective cation channels** (Figure 1A) are perhaps the best-known family of excitatory opsins. Non-selective cation channels allow passage of Na^+^, K^+^, H^+^ and Ca^2+^ along their concentration gradients, depolarizing the cell. In large part, the non-selective cation channel opsins stem from the naturally occurring channelrhodopsin-2 (ChR2) found in *Chlamydomonas reinhardtii*. To optimize ChR2 for use in manipulating mammalian neurons, several variants were made using point mutations. For instance, ChR2(H134R; Nagel et al., 2003), which has an H to R mutation at site 134, is widely used for neuronal manipulation as it displays larger photocurrents than the naturally occurring ChR2 due to an increase in light sensitivity and slower channel closing (Nagel et al., 2003). While the slower channel kinetics increase photocurrent amplitude, they also make ChR2(H134R) less temporally precise than wild-type ChR2. ChR2(H134R) is the most commonly used variant and is referred to simply as ChR2 throughout this review. Several other variants have also been created including: ChIEF (has increased steady-state responses and is able to generate action potentials with high fidelity with high-frequency light stimulation (Lin et al., 2009)); ChETA (has faster kinetics, but reduced photocurrent amplitudes (Gunaydin et al., 2010)); ChR2(ET/TC; has fast activation and deactivation kinetics and is able to generate action potentials with high fidelity at high stimulation frequencies across a wide range of light intensities (Berndt et al., 2011)); and the ChR2(C128X) mutations ChR2(C128A), ChR2(C128T) and ChR2(C128S; Berndt et al., 2009). These ChR2(C128X) mutations show delayed channel closure. Of these mutations, ChR2(C128S) showed photocurrents most similar to the wild-type ChR2 and had the most delayed channel closure. The ChR2(C128S) variant was further combined with a ChR2(D126A; Bamann et al., 2010) variant to produce an even more long-lasting bi-stable step-function opsin (more commonly known as the Stabilized Step Function Opsin (SSFO; Yizhar et al., 2011a). Neurons expressing SSFO display extended depolarization upon activation of the SSFO with a pulse of blue light. A second pulse of light, at a different wavelength (590 nm), is then used to deactivate the SSFO and return the expressing neurons to baseline levels of activity. Most non-selective cation channels related to the naturally occurring ChR2 are maximally activated by blue light at wavelengths of approximately 470 nm. One limitation of these blue-light activated opsins is that blue light has a relatively short wavelength and does not travel far through tissue. High power light delivery can heat tissue, having unintended consequences on physiology, including causing tissue damage at higher levels. In order to overcome this issue, several red-shifted channelrhodopsin variants have been developed/discovered including VChR1 (Zhang et al., 2008), C1V1 (Yizhar et al., 2011b), ReaChR (Lin et al., 2013) and Chrimson (Klapoetke et al., 2014). These red-shifted opsins can be activated with light delivered at lower powers with negligible heating effects from distances further from the target site. Indeed, both ReaChR and Chrimson have been shown to be capable of being activated transcranially in deep brain structures of rodents (Lin et al., 2013; Klapoetke et al., 2014). Recently, an additional transcranial optogenetic method was published, which was based on an alternate method of light delivery, rather than the development of a new opsin; near-infrared light was delivered transcranially and was then absorbed by previously injected upconversion nanoparticles in deep brain structures, that then locally converted the near-infrared photons into blue wavelengths, activating ChR2 (Chen et al., 2018). This tool (lanthanide-doped upconversion nanoparticles) has also been modified to emit green wavelengths (by codoping Er^3+^ and Yb^3+^ into the NaYF_4_ host lattice), capable of activating HR and Arch. These green-light emitting upconversion nanoparticles were used *in vivo* to inhibit acute, chemically induced, seizure activity (Table 2; Chen et al., 2018). Currently the nanoparticles need to be injected into the targeted brain region, preventing this technique from being used in a purely non-invasive manner. While the study examined up to a month past injection, the longer-term stability of the nanoparticles and effects of exposure to the upconversion nanoparticles remains to be studied (Chen et al., 2018). Additionally, if high levels of local blue light are achieved, a potential for tissue heating remains. Regardless of the light source, with the use of any non-selective cation channel opsin like ChR2, which also allow the passage of Ca^2+^, an additional aspect to consider is that the entry of Ca^2+^ into the cell may have unintended consequences on the cell’s physiology.

**The inward chloride pumps** (Figure 1B) are perhaps the best-known family of inhibitory opsins. As the name indicates, this family of opsins actively pumps chloride ions into the cell, thus increasing the intracellular chloride concentration, hyperpolarizing the intracellular space, and lowering the probability of action potential generation. As they actively pump chloride into the cell, chloride pumps can (temporarily) alter the intracellular chloride concentration (Alfonsa et al., 2015), an experimental caveat to be considered when using these opsins. On the flip side, as they are pumps, chloride pumps are not dependent on the reversal potential of chloride and can hyperpolarize and inhibit (at least acutely) even in situations where the chloride reversal potential may be altered (as can happen, for example, in epileptic tissue). Several of the inward chloride pump opsins stem from the naturally occurring halorhodopsin (HR) found in *Natronomonas pharaonis* (NpHR). As with ChR2, several variants of this naturally occurring inhibitory opsin were made in order to optimize their use for manipulating mammalian neurons (Gradinaru et al., 2008, 2010). Compared to the naturally occurring NpHR, eNpHR3.0 displays larger photocurrents due to better membrane trafficking, while preserving the resistance to inactivation seen in wild-type NpHR. As eNpHR3.0 is currently the primary inward chloride pump opsin used for research on mammalian neurons, we refer to it elsewhere in this review as simply HR. An additional inward chloride pump named Jaws, which was derived from *Haloarcula salinarum*, has a red-shifted activation spectrum that has also been shown to be effective in inhibiting neuronal activity through the production of large inhibitory photocurrents (Chuong et al., 2014).

**The outward proton pumps** (Figure 1C) are a distinct method for inhibiting neuronal activity. These opsins function through active pumping of protons out of the cell, thus decreasing the concentration of protons within the cell, hyperpolarizing (and alkalizing) the cell and decreasing the likelihood of action potential generation. These outward proton pump opsins stem from the naturally occurring archaerhodopsin-3 (Arch; Chow et al., 2010) found in *Halorubrum sodomense*. Several variants of this opsin have been developed to better allow sensitive manipulation of mammalian neurons, including ArchT (Han et al., 2011), eArch3.0 (Mattis et al., 2012) and eArchT3.0 (Mattis et al., 2012; enhanced versions have been modified to improve trafficking). Other outward proton pumps have also been discovered to work well in mammalian neurons when modified to improve trafficking including eMac3.0 (Chow et al., 2010; Mattis et al., 2012) and eBR (Gradinaru et al., 2010). Of these opsins, eArchT3.0 displays larger inhibitory photocurrents than either wild-type Arch, eNpHR3.0, or eMac3.0 (Mattis et al., 2012). Although eMac3.0 produces smaller inhibitory photocurrents relative to eArchT3.0 and eNpHR3.0, eMac3.0 is activated by green wavelengths of light (photoactivation spectrum not shown) and is sufficiently blue-shifted to allow for dual-optogenetic inhibition, as in Chow et al. (2010). An important experimental consideration in the use of proton pumps is the resulting alteration in pH. Note that extended light activation of proton pumps on presynaptic terminals can actually cause an increase in calcium influx (and thus downstream signaling) and neurotransmitter release via intracellular alkalization (Mahn et al., 2016).

**The G-protein coupled receptor opsins** (Figure 1D) offer methods for manipulating neural activity via intracellular signaling cascades. The family of G-protein coupled receptor opsins are referred to as OptoXRs (Airan et al., 2009), which includes opto-α_1_-AR (which triggers an α-adrenergic receptor-like response through activation of the G_q_ protein), and opto-β_2_-AR (which triggers a β-adrenergic receptor-like response through activation of the G_s_ protein). An important experimental consideration when using OptoXRs is the potential for expression of the exogenous proteins to alter native receptor expression, localization, or coupling to signaling pathways. Similarly, there is a lack of direct control over which signaling pathways (e.g., downstream of the G protein) the OptoXR expressed will preferentially couple.

**The chloride channel opsins** (Figure 1E), similar to inward chloride pumps and outward proton pumps, can be used for optogenetic inhibition of neural activity. The chloride channels allow for passive flow of chloride ions across the membrane, thus typically hyperpolarizing the cell, decreasing membrane resistance, and decreasing the likelihood of action potential generation. Unlike the inhibitory proton and chloride pumps, which only shuttle one ion per photon, chloride channel opsins allow for movement of hundreds of ions per photon, reducing the need for high intensity light delivery and reducing the likelihood of heating effects. Recently, two naturally occurring chloride channel opsins were discovered in the microbe *Guillardia theta*. These are* Guillardia theta* anion channelrhodopsin 1 and 2 (*Gt*ACR1 and *Gt*ACR2, respectively; Govorunova et al., 2015). *Gt*ACR2 has notably large inhibitory photocurrents and high light sensitivity and may therefore be especially useful in a variety of settings. Before the discovery of these naturally occurring inhibitory channel opsins, inhibitory chloride channels were engineered from the naturally occurring cation-selective channelrhodopsins. These include inhibitory C1C2 (iC1C2; Berndt et al., 2014), iC++ and a bi-stable variant SwiChR++ (Berndt et al., 2016), and a slow chloride-conducting channelrhodopsin (Slow ChloC; Wietek et al., 2014). While activation of chloride channel opsins can inhibit action potentials, when activated at synaptic terminals, rather than inhibiting neurotransmitter release, these opsins can actually trigger neurotransmitter release (Mahn et al., 2016). Regarding the importance of different chloride concentrations (even within the same neuron), and therefore the different impact that activation of chloride channel opsins in different locations on a neuron can have, the reader is further directed to a preprint, not yet peer-reviewed, reference that is particularly relevant: (Mahn et al., 2017). It should also be noted that these chloride channel opsins can both affect and be affected by the chloride ion concentration gradient. This is especially notable in the context of epilepsy research (Pathak et al., 2007; Moore et al., 2017; Wang et al., 2017).

There exist additional options for light-based control of chloride channels (and other receptors more broadly) through a system referred to as optogenetic pharmacology (Kramer et al., 2013; Lin et al., 2015; not illustrated). This method allows for receptor subtype-level control of neuronal activity. Experimental considerations when using an optogenetic pharmacology approach include the need to successfully conjugate the photoswitch prior to attempting neuromodulation; the potential for altering expression or localization of endogenous channels (directly or indirectly); the potential for other effects of the expressed channels, including prior to photoswitch conjugation (especially in contexts where the “photoswitch-ready” version of the subunit is expressed in addition to, rather than in place of, the native subunit); having a mix of light-sensitive and light-insensitive channels; and potential off-target effects or conjugation of the photoswitch itself.

**Other tools include** (Figure 1F) several optogenetic methods of altering protein activity. FLIP (Zhou et al., 2012) and LOVTRAP (Wang et al., 2016) are two methods by which one can conditionally activate proteins when light is delivered and then return the protein to an inactive/bound/caged state when light is terminated. These reversibly photo-inducible proteins are not ideal for long-term activation, as extended light delivery may cause negative phototoxic and heating effects, but these systems are well-suited for experiments that require on-demand or short-lived protein activation. The newly developed photocleavable tool PhoCl (Zhang et al., 2017) has been shown useful for long-term light-induced protein activation, including conditional, quick onset activation of Cre-recombinase (Zhang et al., 2017). Opto-SOS (Toettcher et al., 2013) can activate intracellular signaling proteins upon photo-stimulation. Light-inducible transcriptional effectors (LITES; Konermann et al., 2013) and VP-EL222 (Motta-Mena et al., 2014) are photo-sensitive tools for regulating gene transcription.

Additional notable optogenetic related tools not illustrated include eNPAC (Gradinaru et al., 2010), which is a hybrid of both NpHR and ChR2 and allows for excitation and inhibition of the same cell; BLINK1 (Cosentino et al., 2015), which is a blue-light gated potassium channel; and luminopsins (e.g., inhibitory luminopsins, or iLMO; Tung et al., 2015), which are optogenetic probes that genetically encode their own bioluminescent light source which can be activated in response to a chemical substrate.

**Table 1:** A few examples of optogenetics for research in models of neurological disease.

Disease	Optogenetic tools used	Findings in brief	Key reference
Alzheimer’s disease (AD)	Viral delivery of Cre-dependent ChR2 into CA1 of the hippocampus of PV-Cre AD mice	Optogenetically stimulating hippocampal PV cells, thus driving gamma frequency oscillations, reduced levels of amyloid-beta in AD mice	Iaccarino et al. (2016)
Alzheimer’s disease	cFos-mediated expression of ChR2 in dentate gyrus engram cells; oChIEF expressed under the CaMKIIα promoter injected into the entorhinal cortex	Optogenetically activating dentate engram cells rescues context-dependent memories in amnesic early AD mice. Optogenetic potentiation of entorhinal cortex inputs to the dentate gyrus engram cells resulted in long-term rescue of context-dependent recall in amnesic early AD mice.	Roy et al. (2016)
Alzheimer’s disease	ChR2 expressed under the CaMKIIα promoter injected bilaterally into the hippocampus	Increasing neuronal activity (optogenetically or otherwise) increases tau propagation and pathologies in a mouse model of AD	Wu et al. (2016)
Alzheimer’s disease	Stabilized step function opsin expressed under the CaMKIIα promoter in the lateral entorhinal cortex	Increasing neuronal activity (optogenetically or otherwise) increases amyloid-beta pathologies in a mouse model of AD; optogenetic stimulation also produced behavioral seizure phenotypes	Yamamoto et al. (2015)
Ischemia	Stimulation of ChR2(C128S) to increase acidity or ArchT to alkalize astrocytes and Bergmann glia in cerebellar slices and *in vivo*.	Activation of ChR2(C128S) in glia resulted in glial acidification and increased excitotoxicity in Purkinje cells after cerebellar ischemia. Activation of ArchT in glia resulted in glial alkalization and reduced brain damage after ischemia.	Beppu et al. (2014)
Ischemia	ChR2 expressed under the Thy1 promoter; light delivered bilaterally to activate cortical neurons in a variety of regions	Brain connectivity following stroke undergoes widespread changes, with non-uniform changes in connectivity in the peri-infarct regions and decreases in connectivity even in areas distal from the infarct followed by recovery of some connections and sprouting of other compensatory connections	Lim et al. (2014)
Migraine, ischemia	ChR2 expressed under the Thy1 promoter; light delivered transcranially to activate layer V cortical projection neurons	Developed a non-invasive model of cortical spreading depression using transcranial optogenetic stimulation	Houben et al. (2017)
Multiple sclerosis	ChR2 expressed under the Thy1 promoter; light delivered unilaterally to the premotor area to primarily activate layer V cortical projection neurons	Optogenetic activation of premotor neurons at physiologic frequencies elicits oligodendrogenesis, increases myelination within the premotor cortex and subcortical white matter, and improves motor function in the corresponding limb	Gibson et al. (2014)
Parkinson’s disease	HR or ChR2 expressed under the CaMKIIα promoter; ChR2 expressed under the GFAP promoter; ChR2 expressed under the Thy1 promoter. Light delivered to the subthalamic nucleus (STN).	Optogenetic manipulation of the STN reduced disordered movement in parkinsonian mice; this effect was primarily mediated by the cortical layer V projecting afferents to the STN.	Gradinaru et al. (2009)
Parkinson’s disease	Viral delivery of ChR2 or HR under the CaMKIIα promoter into the striatum or directly onto slice cultures.	Stem cell-derived dopaminergic neurons functionally integrate in a Parkinson’s model and form bidirectional connections between grafted dopamine neurons and host cells.	Tønnesen et al. (2011)
Parkinson’s disease	Viral delivery of Cre-dependent ChR2 into the midbrain of DAT-Cre mice; light targeted bilaterally to dopamine axons in the dorsal striatum	Dopaminergic inputs to the striatum perform rapid phasic signaling that is capable of triggering locomotion in mice.	Howe and Dombeck (2016)
Parkinson’s disease	ChR2 expressed under the Chat promoter; light targeted at the striatum	Striatal cholinergic interneurons mediate exaggerated beta oscillations and induce parkinsonian-like motor deficits.	Kondabolu et al. (2016)
Parkinson’s disease	Viral delivery of Cre-dependent ChR2 into the dorsomedial striatum or globus pallidus externa of D1-Cre, PV-Cre, or Lhx6-iCre mice. For non-specific optogenetic manipulation, ChR2 or ArchT were virally delivered under hSyn or CAG promoters, respectively.	Elevating activity of PV+ Globus Pallidus externa (GPe) neurons over that of Lim homeobox 6 (Lhx6) GPe neurons rescues movement in parkinsonian dopamine depleted mice and provides long-term attenuation of pathological basal ganglia activity	Mastro et al. (2017)

**Table 2:** *In vivo* optogenetics for studying epilepsy.

Type of epilepsy	Model(s) used	Optogenetic tools used	Findings in brief	Key reference
Absence epilepsy	Genetic; spontaneous seizures	Optogenetic activation of cerebellar nucleus neurons virally expressing ChR2 under the hSyn promoter	Closed-loop on-demand optogenetic activation of cerebellar nuclei stopped generalized spike-and-wave discharges characteristic of thalamocortical, absence seizures.	Kros et al. (2015)
Absence epilepsy	Genetic; spontaneous seizures	Unilaterally delivered virus carrying HR or SSFO under the CaMKIIα promoter to induce opsin expression in excitatory neurons of the somatosensory thalamus	Optogenetically inducing thalamocortical rebound phasic spiking unilaterally (using HR) induces bilateral spike-wave discharges in epileptic animals, while optogenetically inducing tonic spiking unilaterally (using SSFO) aborts bilateral spike-wave discharges.	Sorokin et al. (2017)
Absence epilepsy and forebrain and brainstem seizures	Systemic pentylenetetrazole; focally applied bicuculline (piriform cortex); gamma butyrolactone; audiogenic seizures in genetically epilepsy prone rats	Unilaterally delivered virus carrying ChR2 under the hSyn promoter to the deep/intermediate layers of the superior colliculus	Optogenetic activation of the deep/intermediate layers of the superior colliculus suppressed behavioral and electrographic seizures originating in the forebrain, brainstem, and thalamocortical seizure networks.	Soper et al. (2016)
Forebrain and brainstem seizures	Systemic pentylenetetrazole; audiogenic seizures in genetically epilepsy prone mice	ChR2 was expressed in serotonergic neurons under the tryptophan hydroxylase 2 promoter; light targeted to the midline dorsal raphe	Optogenetic activation of the dorsal raphe serotonergic neurons for periods immediately prior to seizure initiation reduced the rate of seizure-induced respiratory arrest	Zhang et al. (2018)
Cortical stroke-induced thalamocortical epilepsy	Photothrombosis in the somatosensory cortex; spontaneous seizures	Unilaterally delivered virus carrying HR under the CaMKIIα promoter into the somatosensory thalamus	Closed-loop on-demand optogenetic manipulation of excitatory thalamocortical neurons interrupts cortical seizure activity.	Paz et al. (2013)
Cortical epilepsy	Tetanus toxin in motor cortex; spontaneous seizures	Unilaterally delivered virus carrying eNpHR2.0 under the CaMKIIα promoter into the motor cortex/seizure focus	Optogenetic inhibition of excitatory motor cortex neurons reduces epileptiform activity.	Wykes et al. (2012)
Cortical seizures	4-aminopyridine applied in barrel cortex; acute seizures	Delivered Cre-dependent virus carrying ChETA to the S1 barrel cortex in PV-Cre mice	Optogenetic activation of PV+ cells during ictal activity terminated seizures; optogenetic activation of PV cells during interictal periods-initiated seizures.	Assaf and Schiller (2016)
Cortical seizures	4-aminopyridine applied in somatosensory cortex; acute seizures	ChR2 was expressed in GABAergic neurons under the VGAT promoter; light targeted unilaterally to the somatosensory cortex	Optogenetic activation of GABAergic cells in hyper-excitable somatosensory cortex during interictal periods initiated ictal-like events in a GABA_A_ receptor dependent manner.	Chang et al. (2018)
Cortical seizures	Optogenetically induced seizures in motor cortex	ChR2 was virally expressed in the motor cortex under the CaMKIIα promoter (for seizure induction). For inhibition of different groups of neurons, either a synapsin promoter or PV-Cre, SOM-Cre, VIP-Cre, Emx1-Cre, or Dlxl12b-Cre mice were injected with a Cre-dependent virus carrying eArch3.0	After optogenetic induction of seizures, optogenetic inhibition of VIP+ neurons inhibited seizures, while inhibition of other populations produced mixed results.	Khoshkhoo et al. (2017)
Temporal lobe seizures	Intrahippocampal bicuculline methiodide; acute epileptiform bursting	Unilaterally delivered virus carrying HR under the hSyn promoter into the ventro-posterior hippocampus; light targeted to CA1/CA2 of the hippocampus	Optogenetic inhibition of hippocampal cells attenuates epileptiform activity in presence of GABA_A_ receptor antagonist.	Berglind et al. (2014)
Temporal lobe seizures	Intraperitoneal kainate, anesthetized animals	Delivered Cre-dependent virus carrying eArch3.0 into the hippocampus of CaMKIIα-Cre mice followed by injection of green light-emitting upconversion nanoparticles into the hippocampus; near-infrared light was delivered transcranially.	Upconversion nanoparticle-mediated optogenetic inhibition of neural activity reduced c-Fos immunoreactivity in granule cells of the dentate gyrus following kainate administration.	Chen et al. (2018)
Temporal lobe epilepsy	Intrahippocampal kainate; spontaneous seizures	Delivered Cre-dependent virus carrying ArchT contralateral to prior WGA-Cre injection to selectively express the opsin in unilateral dentate gyrus mossy cells. In other experiments, Crlc-Cre mice were injected with Cre-dependent virus carrying either ChR2 or eNpHR3.0	Closed-loop on-demand optogenetic excitation of mossy cells ipsilateral or contralateral to the presumed seizure focus reduces behavioral seizure frequency.	Bui et al. (2018)
Temporal lobe seizures	4-aminopyridine applied unilaterally in CA3 of hippocampus; acute seizures	ChR2 was expressed under the Thy1 promoter in transgenic mice; light was delivered unilaterally to the seizure focus (CA3 of the hippocampus)	High frequency optogenetic activation of ChR2-expressing neurons (primarily inhibitory) can provide transient seizure control.	Chiang et al. (2014)
Temporal lobe epilepsy	Intrahippocampal kainate; spontaneous seizures	HR was Cre-dependently expressed by crossing Ai39 mice with CaMKIIα-Cre mice; ChR2 was Cre-dependently expressed by crossing Ai32 mice with PV-Cre mice; light targeted to the hippocampus	Closed-loop on-demand optogenetic inhibition of hippocampal principal neurons ipsilateral or activation of PV+ inhibitory neurons ipsilateral or contralateral to the presumed seizure focus inhibits seizures	Krook-Magnuson et al. (2013)
Temporal lobe epilepsy	Intrahippocampal kainate; spontaneous seizures	ChR2 was Cre-dependently expressed in PV+ neurons by crossing Ai32 mice with PV-Cre mice, or, to more specifically target Purkinje cells, PcP2-Cre mice; light targeted to midline or lateral cerebellum	Closed-loop on-demand optogenetic activation of PV+ neurons, including Purkinje cells, in the lateral cerebellum decreases hippocampal seizure duration, and activation in the midline cerebellum decreases seizure duration and reduces seizure frequency.	Krook-Magnuson et al. (2014b)
Temporal lobe epilepsy	Intrahippocampal kainate; spontaneous seizures	ChR2 or HR was Cre-dependently expressed in dentate gyrus granule cells by crossing Ai32 or Ai39 mice with POMC-Cre mice, respectively	Activation of dentate gyrus granule cells in epileptic (or naïve) mice can induce or worsen seizures. Closed-loop on-demand inhibition of granule cells can efficiently stop seizures.	Krook-Magnuson et al. (2015a)
Temporal lobe seizures	4-aminopyridine applied unilaterally in CA3 of hippocampus; acute seizures	Used transgenic mice expressing ChR2 under the Thy1 promoter; and to specifically target inhibitory neurons, used a transgenic mouse line expressing ChR2 under the VGAT promoter	Low frequency optogenetic stimulation of hippocampal neurons reduced seizure activity.	Ladas et al. (2015)
Temporal lobe seizures	Intrahippocampal kainate; intra-amygdala kainate; acute seizures	ChR2 expression under the VGAT promoter in a transgenic mouse line; delivered Cre-dependent ChR2 or HR virally into the dentate gyrus of GAD-Cre mice	Optogenetic activation of inhibitory neurons in the dentate gyrus suppresses ictal activity in the dentate and in the entorhinal cortex. Optogenetic activation of inhibitory neurons in the entorhinal cortex inhibits ictal activity in the entorhinal cortex but not dentate.	Lu et al. (2016)
Temporal lobe seizures	Optogenetically evoked hippocampal seizures	ChR2 expressed under a Thy1.2 promoter in transgenic rats; ChR2 expressed under a beta-actin promoter; light delivered to the hippocampus, thalamus, amygdala, and sensorimotor cortex	Repetitive pulse photostimulation to the opsin-expressing hippocampus induced seizure-like afterdischarges that were sustained and propagated along the longitudinal axis of the hippocampus.	Osawa et al. (2013)
Temporal lobe seizures	Lithium-pilocarpine elicited seizures; acute seizures	Unilaterally delivered virus carrying HR under the CaMKIIα promoter into the hippocampus	Optogenetic inhibition of hippocampal pyramidal cells prior to seizure onset delayed initiation of status epilepticus and altered seizure development.	Sukhotinsky et al. (2013)
Temporal lobe seizures	Optogenetically evoked hippocampal seizures	Unilaterally delivered virus carrying ChR2 under the CaMKIIα promoter into the dorsal or intermediate hippocampus	High-frequency optogenetic activation of principal cells in the intermediate hippocampus during fMRI showed recruitment of widespread cortical and subcortical networks.	Weitz et al. (2015)
Temporal lobe seizures	Electrical hippocampal kindling	Used a transgenic mouse line expressing ChR2 under the VGAT promoter to target inhibitory neurons; or viral delivery of Cre-dependent ChR2 in mice expressing Cre in PV+ or SOM+ cells; or viral delivery of Cre-dependent Arch or HR in mice expressing Cre under the CaMKIIα promoter to inhibit excitatory cells; light delivered to the subiculum or CA3.	Activation of GABAergic neurons, or direct inhibition of excitatory cells with HR or Arch, in the subiculum delayed acquisition of secondary generalized seizures during kindling. In fully kindled animals, activation of subicular PV+ cells had a pro-ictal effect while activation of SOM+ cells inhibited ictal activity. While activation of inhibitory neurons in the subiculum had a pro-ictal effect in kindled animals, activation of inhibitory neurons in CA3 inhibited ictal activity. In kindled animals, direct inhibition of excitatory subicular cells with Arch, but not HR, inhibited ictal activity.	Wang et al. (2017)
Temporal lobe, forebrain and brainstem seizures	Intrahippocampal bicuculline methiodide; systemic pentylenetetrazole; acute seizures	Bilaterally delivered virus carrying the inhibitory luminopsin (iLMO) under the CaMKIIα promoter into the dentate gyrus and/or the anterior nucleus of the thalamus (ANT)	Activation of iLMO in the principal cells of the dentate gyrus or glutamatergic neurons of the ANT prior to the seizure delayed seizure onset and reduced seizure duration. Activation of iLMO in both the dentate gyrus and, simultaneously, the ANT had an additive effect, reducing seizure duration and severity.	Tung et al. (2018)

Box 2 **Achieving opsin expression.**

Opsins are derived from naturally occurring light-sensitive proteins found in various archaea and algal species. There is an ever-expanding toolbox of opsins and other light-sensitive proteins with distinct properties and unique downstream effects on the cells that express them (Box 1, Figure 1). This versatility is one of the features that make optogenetic tools so useful for neuroscience research. However, an early challenge to utilizing opsins’ power and versatility broadly in a neuroscience research setting was successfully achieving opsin expression in mammalian neurons. In 2005 the excitatory opsin, channelrhodopsin-2, was successfully expressed in cultured hippocampal neurons using a lentiviral gene delivery strategy (Boyden et al., 2005). This study demonstrated the viability of optogenetics for neuroscience and laid the foundation from which an entire field of study has grown (Boyden, 2015; Deisseroth, 2015).

When discussing the advantages of optogenetics over various other approaches, one must emphasize its specificity; optogenetics allows for bi-directional neuronal manipulation with incredible temporal, spatial, and cell-type specificity. Opsins are activated by light, allowing for potentially great temporal and spatial specificity, as light can be quickly delivered to a highly restricted and well-defined spatial location with relative ease. Opsins with rapid activation and deactivation kinetics have been engineered to allow fine-grained control over neuronal output (Box 1). However, opsins are not inherently cell-type specific. This is actually a strength and a trait that helps make opsins so versatile: with the proper gene delivery system, opsins can be expressed in virtually any cell population. For instance, opsins can be directly expressed under a general promoter, a pan-neuronal, or glial promoter. Importantly, more selective expression can also be achieved. Indeed, not only have new opsins and other genetically-encoded light-sensitive tools been developed over the past several years (Box 1), but methods for achieving specific expression have also improved greatly. The advancements to methods for achieving selective opsin expression (especially in mice) have given neuroscientists the ability to study specific cell populations’ activity *in vitro* and *in vivo* with relative speed and ease. We discuss in this box some current methods used to achieve selective opsin expression.

Opsins may be expressed directly under a promoter—as mentioned above, this promoter can be a broad or general promoter. If, however, more restricted expression is desired, a cell-type specific promoter can be used. There are a number of transgenic lines that utilize this approach. A cell-type specific promoter can also be used with a viral vector approach. The CaMKIIα promoter is one often used in this context (including, for example, several publications listed in Tables 1, 2). Using a cell-type specific promoter based approach has some major limitations, however. First, especially with a viral vector approach, leaky expression in other cell populations is sometimes reported. Second, if the promoter under which the opsin is to be expressed is long, it may not fit into commonly used vectors. And third, if the promoter of interest is a weak promoter, opsin expression will also be weak. To circumvent these limitations without sacrificing the ability to target opsin expression to a select population of cells, a Cre/loxP mediated approach is often taken (Figure 3). With such an approach, the opsin can be placed under a strong general promoter, but expression is made to be Cre-dependent.

There are several ways to achieve Cre-dependent opsin expression. A segment of DNA located between two loxP sites (i.e., a segment that is floxed) will be either irreversibly excised or reversibly inverted by Cre recombinase, depending on the relative orientation of the loxP sites. One method to achieve Cre-dependent opsin expression utilizing DNA excision is to insert a floxed STOP cassette upstream which prevents the opsin from being expressed until Cre-mediated excision of the STOP cassette (Figure 3Ai). This approach works well in transgenic mouse lines to restrict expression (Madisen et al., 2012). Because the excision of the STOP cassette is permanent, and the opsin itself is placed under a strong promoter, even weak levels of Cre-expression can be sufficient for robust opsin expression. This can circumvent issues arising due to cell-type specific promoters being weak or inconsistently expressed over time. It also can lead to difficulties, for example if a promoter is transiently used by a different cell population or during development. This is a caveat to consider for all recombination-based methods for selective expression.

Theoretically, a floxed STOP cassette could also be implemented in a viral vector, rather than solely in transgenic animals to achieve Cre-dependent opsin expression. This method was found to lead to leaky opsin expression, however. To overcome this limitation, another method for achieving Cre-dependent opsin expression in viral vectors is to place the opsin in the anti-sense direction between two loxP sites oriented such that Cre will invert the DNA between them, thus putting the opsin in the sense direction and allowing expression. Alone this method provides weak opsin expression, as the opsin can be re-inverted back to the anti-sense direction. However, when combined with a second set of mutated loxP sites, the inversion can be made irreversible (see Figure 3Aii). This method, referred to as a FLEX (flip-excision; Atasoy et al., 2008) or double-floxed inverse open reading frame (DOI; Zhang et al., 2010) is now commonly used to attain specific and strong opsin expression with viral-based gene delivery.

For all Cre-based approaches, Cre-expression can be achieved through a (second) transgenic line or through viral-based methods (Figure 3B). In some circumstances, viral vector-based methods can result in un-even or over-expression of proteins, evoke an immune response, or show diminished effectiveness due to an immune response. However, viral vector approaches provide an important compliment to purely transgenic approaches.

When vectors that produce retrograde labeling^1^ are used, viral based methods for Cre or opsin expression can also be used to gain selective expression in cell populations not defined by a neurochemical marker but instead by a projection target (Figure 6). There are a number of potential strategies here too, including the use of modified rabies (Wall et al., 2010; Sun et al., 2014; Chatterjee et al., 2018), canine adenovirus-2 (CAV; Junyent and Kremer, 2015; Namburi et al., 2015), herpes simplex virus (HSV; Fenno et al., 2014), engineered adeno-associated virus (AAV) AAV2-retro (Tervo et al., 2016), or Cre fused to wheat germ agglutinin (WGA-Cre; Gradinaru et al., 2010; Libbrecht et al., 2017). The use of WGA-Cre to achieve selective opsin expression recently allowed the examination of the role of mossy cells in temporal lobe seizure evolution (Bui et al., 2018; Table 2). Additionally, to overcome the limitation of cytotoxicity caused by previous rabies approaches, a class of relatively non-toxic rabies viral vectors have been developed (Chatterjee et al., 2018).

So far, all of the methods described above allow for expression of an opsin in a cell population as defined by one feature: for example, expression of a neurochemical marker (such as parvalbumin) or, in the case of target-based labeling, projection to a specific region. Cells are often defined by multiple characteristics, however, and distinct cell populations can share a given neurochemical marker or other partially defining feature. Recently, an approach to achieve opsin expression based on two features (rather than one) was developed. This intersectional approach extends the principles of the FLEX/DIO system described above and uses both the Cre/loxP and Flp/Frt recombination systems to limit expression (Figure 3Aiii). These cleverly designed viral vectors, known as INTRSECT, allow Boolean-logic based approaches to limit expression to cells that express both Cre *and* Flp (Con/Fon), or to cells that express Cre and *not* Flp (Con/Foff), or to cells that express Flp and *not* Cre (Coff/Fon). Intersectional optogenetic approaches allow for improved cell-type specificity and greatly improve the versatility and power of optogenetics for use in unraveling complex neuronal circuits.

The tools available to achieve selective opsin expression are, and hopefully will continue to be, rapidly expanding. Through selective opsin expression, selective neuromodulation is possible, and thereby we can gain greater insight into neurological circuits in health and disease.

**Figure 4:**
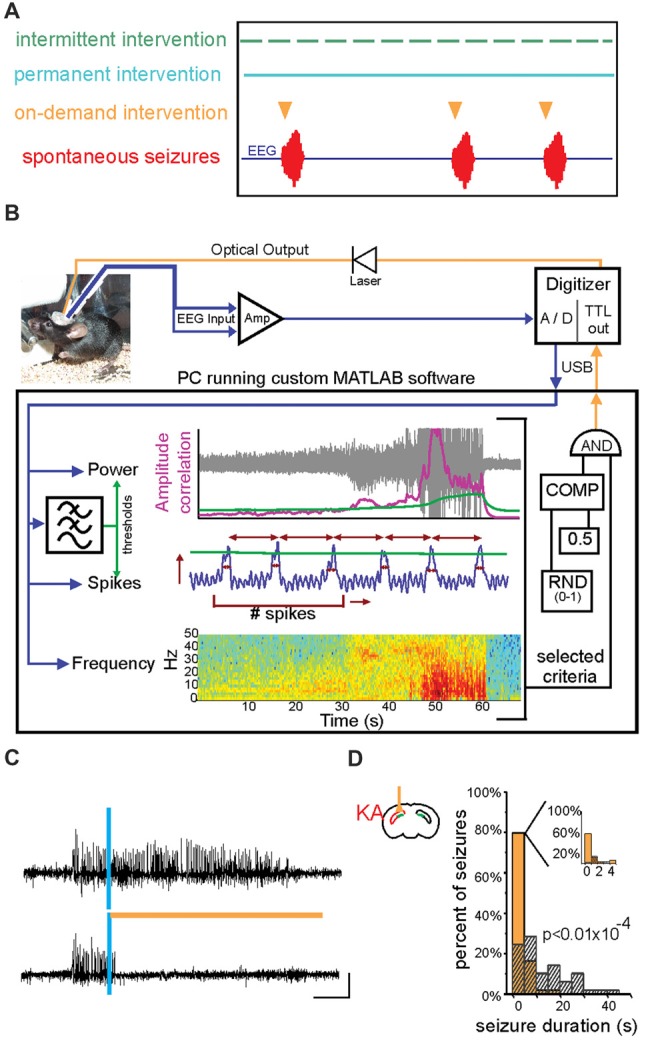
On-demand and closed-loop seizure intervention strategies. **(A)** Options for the timing of various seizure interventions illustrated relative to spontaneous seizure events (in red, bottom trace). Traditional seizure treatment options, including pharmacological and some neuromodulatory approaches, fall within the intermittent intervention strategy in which the therapy is delivered on a prescribed schedule irrespective of seizure timing (green). Other intervention strategies, such as surgical resection of the seizure focus, are clearly permanent and are also not sensitive to the timing or frequency of seizures (blue). In contrast, on-demand seizure interventions are applied only at the time of seizure prediction/detection and are therefore only used when necessary (orange). This on-demand method of seizure intervention may lead to fewer negative side effects and intervention-related complications. **(B)** An example of a closed-loop system for on-demand optogenetic seizure intervention in mice. EEG signals (blue) are amplified (Amp) and digitized (A/D) and dispatched into custom MATLAB software for real-time analysis of the signal power, frequency, and several spike characteristics (including spike number, width, amplitude, and rate). Threshold levels (green) for these characteristics are selected to achieve accurate seizure detection in each animal. A trigger signal is then sent to the digitizer and on to the LASER to begin a light protocol for a given percentage of detected seizures (in the illustrated system, 50% of detected seizures would be selected in a random fashion (RND) to trigger light delivery). COMP: digital comparator; USB: universal serial bus. **(C)** Representative spontaneous seizures recorded from the hippocampus of a mouse previously injected with kainate (KA) and which expresses HR in granule cells. Time of seizure detection by real-time analysis using custom MATLAB software (see also **B**) illustrated with vertical blue bar. The top trace shows a detected seizure that did not receive light intervention. In contrast, the bottom trace shows a seizure that triggered light delivery (orange horizontal bar). Note that the seizure in the bottom trace is quickly terminated after light delivery. **(D)** Summary data for one mouse showing a histogram of post-detection seizure durations with no light intervention (gray hashed bars) compared to those that did receive light intervention (orange solid bars). In these experiments **(C,D)**, light (orange bar) was delivered ipsilateral to the site of previous KA injection (red in schematic of coronal section). Scale bar (D) = 0.2 mV, 5 s. **(A)** modified with permission after Figure 1A from Krook-Magnuson et al. (2015b) **(B)** from Krook-Magnuson et al. (2013). **(C,D)** modified with permission from Krook-Magnuson et al. (2015a).

**Figure 5:**
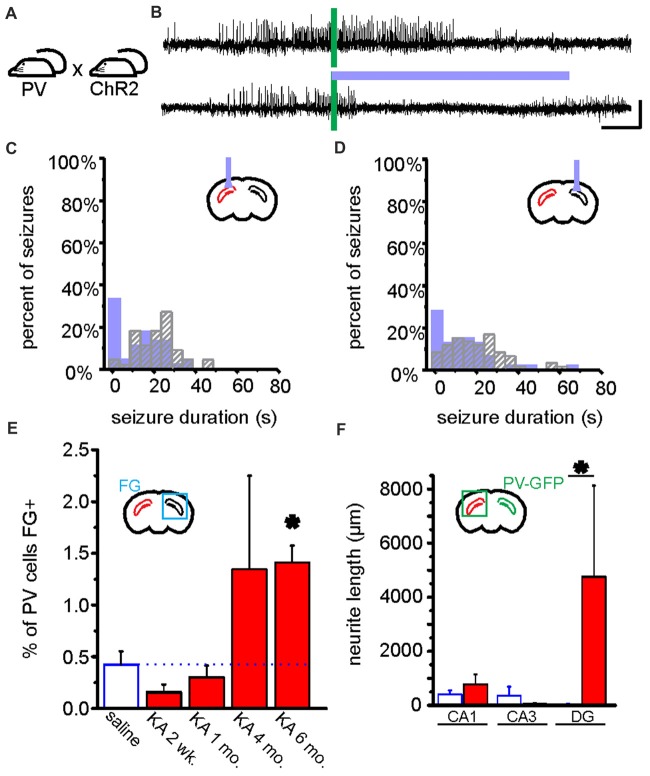
Optogenetic manipulation and circuit dissection of inhibitory PV cells in a mouse model of chronic temporal lobe epilepsy (TLE). **(A)** PV-Cre mice were crossed with mice expressing ChR2 in a Cre-dependent manner. Offspring of this cross expressed ChR2 selectively in PV cells. These mice received a unilateral intrahippocampal kainate (KA) injection to model chronic TLE. Blue light was targeted to the dorsal CA1 region of the hippocampus for optogenetic activation of hippocampal PV cells. Data from these mice are shown in **(A–D)**. **(B)** An example seizure where no blue light was delivered (top trace), and where blue light was delivered (bottom trace). Green bars indicate time of seizure detection by the closed-loop algorithm (see Figure 4). Scale bars: 5 s, 100 μV. **(C,D)** Post-detection seizure duration of detected seizures where no blue light was delivered (gray hashed bars), and where blue light was delivered (filled bars). In **(C,D)**, see inset schematics of light delivery (purple probe) relative to the hippocampus previously injected with KA (red). On-demand light delivery to activate PV cells increases the percentage of seizures stopping within 5 s of seizure detection, whether light was delivered ipsilateral **(C)** or contralateral **(D)** to the site previously injected with KA. **(E)** After an injection of the retrograde tracer Fluorogold (FG) into the left hippocampus (i.e., the hippocampus previously injected with KA or saline), the PV cells in the right hippocampus were analyzed for FG colocalization either 2 weeks, 1 month, 4 months, or 6 months following KA or saline. In the saline control mice (data collapsed across time points) approximately 0.5% of PV cells in the right hippocampus were colabeled with FG. Two weeks following KA, there was a slight, but non-significant decrease in the percentage of PV cells colabeled with FG followed by a progressive increase (asterisk indicates *P* value of less than 0.01; 6-month KA vs. saline controls). **(F)** Commissurally projecting PV cell axons were visualized and measured in a PV-Cre mouse line injected in the right hippocampus with a Cre-dependent GFP virus. Six months post-KA (red), there are significantly longer axons (more than 100x longer) in the dentate gyrus from commissurally projecting PV cells when compared to the 6-month post-saline controls. **(A–D)** modified with permission after Krook-Magnuson et al. (2013). **(E,F)** modified with permission after Christenson Wick et al. (2017).

**Figure 6:**
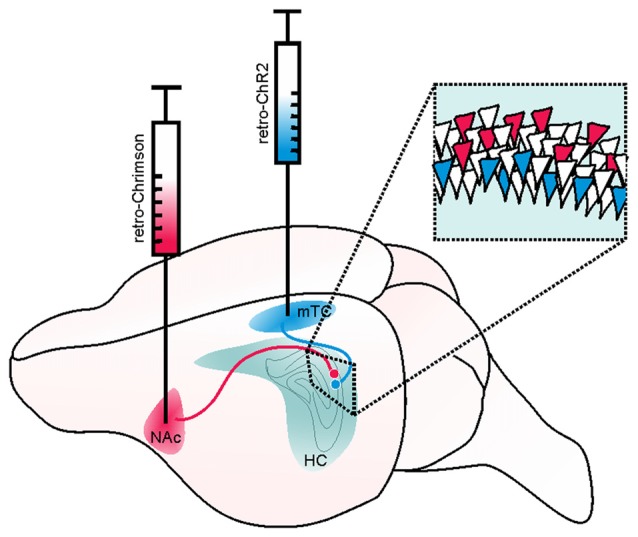
Example of a retrograde viral vector-based approach to selectively target deep and superficial pyramidal cells of the hippocampus (HC). Retrograde viral vector-based approaches to opsin delivery (such as WGA-Cre, AAV2-retro, or CAV) may be used to independently target and manipulate cells with distinct projection targets. In the illustrated hypothetical example, this method is used to separately target and manipulate deep and superficial pyramidal cells of the hippocampus in the same mouse. Such an approach could be used, for example, to test the hypothesis that deep and superficial pyramidal cells may contribute uniquely to hippocampal seizures or their spread. Superficial CA1 pyramidal cells (that is, those on the stratum radiatum side of the pyramidal cell layer) project to the medial temporal cortex (mTC) while deep pyramidal cells project to the nucleus accumbens (NAc). Therefore, by injecting a retrogradely transported viral vector carrying a blue-light activated opsin (e.g., ChR2, shown in blue) into the mTC, one could selectively target and manipulate superficial (and not deep) pyramidal cells. Likewise, by injecting a retrogradely transported viral vector carrying a red-shifted opsin (e.g., Chrimson, shown in red) into the NAc, one could selectively target and manipulate deep pyramidal cells. As these opsins have sufficiently distinct activation spectra, the two populations of neurons could be independently manipulated in the same animal. Inset illustrates a portion of stratum pyramidale in CA1 of the hippocampus, with schematic expression of ChR2 (blue) in superficial and Chrimson (red) in deep pyramidal cells.

Optogenetics thereby gives researchers an unprecedented ability to manipulate neuronal populations with cell type, spatial, directional and temporal specificity. This specificity makes optogenetics a powerful tool. Adding to its success, optogenetic techniques can be relatively easy to implement, in large part due to active decisions by tool developers to make tools widely accessible. Importantly, optogenetics is also a flexible platform, which can be adapted to the experimenter’s research needs. The continual creation and application of new optogenetic related tools further expands the usefulness of optogenetics in neuroscience research. Given these strengths (specificity, versatility, and continual development) of optogenetic approaches, it is not surprising that optogenetics is being rapidly embraced by a variety of neuroscience fields (Deisseroth, 2015; Montgomery et al., 2016).

Optogenetics has proven to be a highly powerful and useful tool for studying healthy and pathological brain activity from a cellular and systems/circuit level perspective (Boyden, 2015; Deisseroth, 2015). Indeed, optogenetics has become so ubiquitous a tool for studying the brain that it is not feasible to describe all of its uses and contributions in a single review. To illustrate the broad versatility and power of optogenetics, Table 1 highlights a few key examples of optogenetics being used to study neurological disorders that the World Health Organization has named among the most burdensome. One such neurological condition is epilepsy (Table 2; Krook-Magnuson and Soltesz, 2015).

Epilepsy is the fourth most common neurological disorder, affecting roughly 65 million people worldwide (England et al., 2012). Unfortunately, in large part due to a critical lack of understanding of many fundamental aspects of epilepsy, current treatment options fall short. It is estimated that one third or more of epilepsy patients do not achieve adequate seizure control with current treatment options, and current treatment options can have major negative side effects (de Tisi et al., 2011; Perucca and Gilliam, 2012; Duchowny and Bhatia, 2014; Laxer et al., 2014). Optogenetics, by providing researchers with a powerful and flexible tool, is allowing significant advances in our understanding of epilepsy (for additional reviews related to the general topic of epilepsy and optogenetics, see also Krook-Magnuson et al., 2014a; Krook-Magnuson and Soltesz, 2015; Tung et al., 2016; Choy et al., 2017; Forcelli, 2017; Tønnesen and Kokaia, 2017). Here, we discuss this work in epilepsy research highlighting how well-suited an optogenetic approach is to epilepsy research in particular and how the benefits of an optogenetic approach can be leveraged in future research. Optogenetic techniques have been uniquely valuable in identifying and testing brain circuit elements that may be capable of seizure control, testing hypotheses related to epilepsy, and determining the roles of specific neuronal populations in epilepsy and seizures.

While optogenetics is a powerful tool in neuroscience research, there are a number of practical hurdles which must be overcome before optogenetics can be used in a clinical setting for epilepsy or other neurological disorders. As discussed in more depth elsewhere (Kullmann et al., 2014; Krook-Magnuson and Soltesz, 2015), these challenges include demonstrating safety of opsin expression, determining the optimal methods to achieve opsin expression in human tissue, and devices for appropriate light delivery. To date, optogenetic techniques have had the most success in smaller animals, including rodents (see e.g., Tables 1, 2), zebrafish (e.g., Motta-Mena et al., 2014; Cosentino et al., 2015; Kainrath et al., 2017), and *Drosophila* (e.g., Zemelman et al., 2002; Dawydow et al., 2014; Liu et al., 2017; Mohammad et al., 2017). The “scaling up” of optogenetic techniques to larger animals is an additional potential issue which needs to be addressed; however, recent advancements in this arena have been made (e.g., Yazdan-Shahmorad et al., 2018). Despite these challenges, optogenetics may one day be used not only as a research tool, but also as a clinical tool. Optogenetic based approaches are currently entering early clinical trials for treating retinitis pigmentosa through companies including GenSight Biologics (Paris, France) and RetroSense Therapeutics (Ann Arbor, MI, USA). If these trials demonstrate that exogenous opsins can be safely expressed in humans, further investigation of the clinical potential of optogenetics can be expected. Epilepsy is likely to be one of the subsequent early targets for the clinical application of optogenetics. This is due to: (i) the success of some early preclinical optogenetic work in the epilepsy field discussed in part below; (ii) the opportunity for optogenetics to fill a clinical need for new treatment strategies for epilepsy that are more efficacious with fewer negative side effects; and (iii) practical reasons, as one current treatment strategy for epilepsy is surgical removal of brain tissue. Thus, an optogenetic approach could be tried prior to surgical resection, with opsin expression attempted in the brain area that would otherwise be removed; if the optogenetic approach failed, one could then proceed with tissue removal. A caveat to this, however, is if opsin expression were to spread outside of the would-be-transected region, for example through retrograde transport of the viral vector (Kaemmerer, 2017). Importantly, even if optogenetics itself does not reach the clinic, the insight gained through research using optogenetic methods is certain to have a beneficial impact on patient care (Rajasethupathy et al., 2016).

Using epilepsy research as a focal point, we discuss in turn examples of how the specificity of optogenetics can be harnessed; the diverse ways in which optogenetics can be used in research; and finally, how newly developed and future tools can aid in addressing outstanding questions in the field.

## Specificity

One of the major benefits of an optogenetic approach is the level of specificity of intervention one can achieve. Therapeutically, an intervention strategy which is highly restricted may be able to avoid unnecessary negative side effects and intervention-related complications. Therefore, one of the first epilepsy research questions tested using this tool was whether optogenetics—a highly restricted intervention strategy—could also be an *effective* intervention strategy. Epilepsy is often referred to as a network or circuit disorder (Bernhardt et al., 2015; Jin et al., 2015; Vaughan et al., 2016) to emphasize the importance of neuronal interactions in seizures. In part because of this “network” view, it was unclear if a highly restricted intervention strategy (targeting one cell type in one region) could effectively inhibit seizures. This fundamental question was tested through a variety of experimental approaches, and optogenetics was ultimately shown to be effective at inhibiting: (i) epileptiform activity *in vitro* (Tønnesen et al., 2009; Berglind et al., 2014; Sessolo et al., 2015); (ii) induced, acute seizures in non-epileptic animals (Sukhotinsky et al., 2013; Berglind et al., 2014; Chiang et al., 2014; Ladas et al., 2015; Lu et al., 2016; Soper et al., 2016); and (iii) spontaneous seizures in various rodent models of different forms of epilepsy (Wykes et al., 2012; Krook-Magnuson et al., 2013; Krook-Magnuson et al., 2014b; Krook-Magnuson et al., 2015a; Paz et al., 2013; Kros et al., 2015; Sorokin et al., 2017; Bui et al., 2018). Table 2 provides an overview of studies using *in vivo* optogenetics in the field of epilepsy research. Importantly, a number of studies have harnessed not only the spatial specificity and cell-type specificity of optogenetics, but also the tool’s temporal specificity by implementing a closed-loop approach (Figure 4; Armstrong et al., 2013; Grosenick et al., 2015; Krook-Magnuson et al., 2015b; Nagaraj et al., 2015). In these studies, on-line seizure detection allowed light delivery to be restricted to times of spontaneous seizures (referred to as on-demand optogenetics), providing an additional element of specificity to the interventions (Krook-Magnuson et al., 2013; Krook-Magnuson et al., 2014b; Krook-Magnuson et al., 2015a; Paz et al., 2013; Sorokin et al., 2017; Bui et al., 2018).

### Optogenetic Neuromodulation Allows More Direct Testing of Hypotheses Through Selective Perturbation

The specificity of intervention possible through on-demand optogenetic techniques also provides an important research tool to test additional hypotheses related to epilepsy. For example, in the field of temporal lobe epilepsy (TLE) research there is a long-standing hypothesis called “the dentate gate hypothesis” (Heinemann et al., 1992; Lothman et al., 1992). In healthy control tissue, the dentate gyrus is thought to act as a gate; due to intrinsic and circuit level properties, dentate gyrus granule cells only show very sparse activation in response to entorhinal cortex input. The dentate gate hypothesis posits that, in TLE, there is a breakdown of this gating function, granule cells become relatively over-active, and too much excitation flows through the hippocampal circuit. Whether the excitatory drive pushing seizures arrives from the entorhinal cortex, some other outside source, or from the dentate itself is currently under consideration (Meyer et al., 2016; Krook-Magnuson, 2017). Regardless of the source of the excitatory drive, fundamental to the dentate gate hypothesis is that granule cells show heightened activity levels. Supporting the dentate gate hypothesis, there are a number of changes in epileptic tissue that would reduce inhibition and increase excitation of dentate gyrus granule cells (Nadler et al., 1980; Coulter, 1999; Bouilleret et al., 2000; Zhang et al., 2012).

While there is abundant correlative data showing an increase in dentate excitability in TLE, until recently, strong evidence indicating causality (rather than merely correlation) to support the dentate gate hypothesis was lacking; prior to the advent of optogenetic techniques, it was difficult to directly test the dentate gate hypothesis. With on-demand optogenetic techniques however, researchers were able to test causality rather than just correlation by artificially causing a breakdown of the dentate gate (by optogenetically exciting granule cells) and by “restoring” the dentate gate (by optogenetically inhibiting granule cells) at the time of spontaneous seizures in epileptic animals (Krook-Magnuson et al., 2015a; Table 2). In doing so, researchers took advantage of: (i) the cell type specificity (targeting just granule cells); (ii) the temporal specificity (restoring the gate selectively at the time of seizures); and (iii) the control over the direction of modulation (excitation vs. inhibition) achievable with optogenetic approaches. Additionally, the authors examined whether the location of inhibition or excitation of granule cells mattered by delivering light either ipsilateral or contralateral to where the “break-down of the gate” was thought to occur.

Supporting the dentate gate hypothesis, the authors found that on-demand optogenetic excitation of granule cells (“flooding the gate”) worsened seizures in epileptic animals and even induced seizures in non-epileptic animals (Krook-Magnuson et al., 2015a). These findings also highlight that optogenetics can be used not only to inhibit seizures, but also to induce seizures. Optogenetic induction of seizures is not unique to activation of granule cells, and several studies have used optogenetic approaches to induce seizures (Osawa et al., 2013; Weitz et al., 2015; Khoshkhoo et al., 2017; Table 2). However, activation of the dentate may still be fairly distinct in regard to generating seizures: it was recently reported that optogenetic activation of neurons to produce cortical seizures requires increasingly stronger activations, with subsequent light deliveries, in order to continue to produce seizures (Khoshkhoo et al., 2017; Table 2). In contrast, optogenetic excitation of dentate granule cells in control animals displayed a rapid kindling effect, such that each subsequent light delivery produced a larger, more severe, seizure (Krook-Magnuson et al., 2015a). Providing further support for the dentate gate hypothesis, using non-optogenetic methods to increase the excitability of dentate granule cells is also sufficient to induce epilepsy (Pun et al., 2012).

Perhaps the greatest optogenetic support for the dentate gate hypothesis, however, comes from the experiments which have not induced seizures, but rather inhibited seizures in chronically epileptic mice. Experimenters used optogenetics to *restore* the dentate gate by selectively inhibiting granule cells at the time of seizures. When this optogenetic restoration of the dentate gate occurred ipsilateral to the presumed seizure focus (i.e., where the breakdown of the gate would be expected to occur), focal seizures were immediately inhibited (Figures 4C,D). Interestingly, and in-line with the hypothesis, inhibition of granule cells in the contralateral dentate gyrus (where the native gate should still be functioning) had no effect on the seizures.

Together, the findings provide strong support for the dentate gate hypothesis and illustrate the power of using the cell-type selective and temporally precise neuromodulation achievable with on-demand optogenetic methods. It also demonstrates the importance of direct control of the direction of modulation (excitation vs. inhibition), which can sometimes be difficult with electrical stimulation methods. Finally, the seizure inhibition achieved by selectively inhibiting granule cells was comparable to the level of seizure inhibition achieved previously by broadly inhibiting excitatory cells in the hippocampal formation (Krook-Magnuson et al., 2013; Table 2). Therefore, these findings further illustrate that identifying key components of the network (in this case, dentate gyrus granule cells) can allow refinement of an intervention without sacrificing efficacy.

An additional example of using the specificity of *in vivo* on-demand optogenetics to test hypotheses in epilepsy research is provided by work conducted by Paz and colleagues (Table 2). Paz et al. (2013) found that after an experimentally induced cortical stroke, rats developed epilepsy. The researchers hypothesized that cortical stroke was inducing thalamocortical epilepsy (i.e., that the thalamus was critically engaged during these seizures). To test this hypothesis, Paz et al. (2013) used a viral vector with a CaMKIIα promoter to express the inhibitory chloride pump halorhodopsin (HR) in the ventrobasal thalamus ipsilateral to the cortical stroke. Light was then delivered in an on-demand fashion to inhibit thalamic neurons. Seizures were truncated by this approach, supporting the importance of the thalamus in cortical stroke-induced epilepsy.

More recently, Paz and colleagues have used optogenetic methods to further demonstrate that the firing pattern of thalamocortical cells is critically important for seizure control (Sorokin et al., 2017; Table 2). Rather than briefly inhibiting thalamocortical neurons in an on-demand manner at the time of seizures as done previously, the researchers examined the effect of using an *excitatory* stabilized step function opsin (SSFO, see Figure 1A and Box 1) in genetic models of absence epilepsy. Light delivery placed the thalamocortical neurons in a tonic mode of firing, and (just as with brief inhibition in stroke induced seizures) inhibited seizures.

In the same study, Paz and colleagues also examined the effect of inducing burst firing interictally (that is, when the animal was not actively seizing) in animals genetically prone to absence seizures (Sorokin et al., 2017). Using pulses of light to briefly and repeatedly inhibit thalamocortical cells (which have strong T-type calcium channel-mediated currents), researchers were able to induce rebound firing in thalamocortical cells, entrain their firing, and mimic a burst firing mode. Such pulsed light delivery was able to *initiate* absence seizures in these animals. Together, the work by Paz and colleagues indicates that in epileptic animals, manipulation directed at the thalamus is able to induce thalamocortical absence seizures, and that disruption of on-going activity of thalamocortical cells during seizures (either by gentle excitation to induce tonic firing or by brief direct inhibition) can be used to truncate thalamocortical seizures, underscoring the importance of the thalamus in this seizure network.

### The Broader Seizure Network?

The examples discussed above demonstrate the utility of on-demand optogenetics in more directly testing the importance of circuit elements hypothesized to be critically engaged in seizure networks. That is, by directly manipulating the thalamus, Paz and colleagues were able to provide strong support for the causal role the thalamus can play in thalamocortical absence epilepsy (Paz et al., 2013; Sorokin et al., 2017). Likewise, the importance of the dentate gyrus in TLE was demonstrated through direct manipulation of dentate gyrus granule cells (Krook-Magnuson et al., 2015a). Optogenetic methods are also useful in examining circuit elements that are not typically considered to be part of the seizure network but may still be able to provide seizure control. In this context, we will briefly discuss work using optogenetic methods to examine the utility of targeting the cerebellum or deep layers of the superior colliculus for a variety of epilepsies.

The cerebellum is not typically associated with epilepsy, but in large part due to recent work, including optogenetic work, that viewpoint is changing. Specifically, recent studies (Table 2) have shown that on-demand optogenetic manipulation of the cerebellum can inhibit seizures in rodent models of both thalamocortical absence epilepsy (Kros et al., 2015) and TLE (Krook-Magnuson et al., 2014b). Surprisingly, on-demand optogenetic excitation *or* inhibition of the cerebellar cortex was able to inhibit temporal lobe seizures (Krook-Magnuson et al., 2014b), suggesting that simply disrupting cerebellar activity was able to terminate hippocampal seizures. This is perhaps somewhat akin to results examining the effects of manipulation of thalamic cells during thalamocortical seizures discussed above (Paz et al., 2013; Sorokin et al., 2017; Table 2). More recently, Kros et al. (2015) examined the effect of optogenetic activation of the cerebellum (in particular, deep cerebellar nucleus neurons) during absence seizures, and found that on-demand stimulation was able to also abort thalamocortical seizures. Taken together, these results are very exciting, and suggest a potentially broad applicability of targeting the cerebellum in various epilepsies. Such a target with broad applicability would be valuable in treating patients whose seizures have multiple, diffuse, uncertain, or progressing seizure foci—these patient populations are currently very difficult to treat through directed neuromodulation methods.

Interestingly, when Kros et al. (2015) took a pharmacological, rather than optogenetic, approach to manipulate the deep cerebellar nuclei in absence epilepsy, inhibition (through local application of the GABA_A_ receptor agonist muscimol) increased the likelihood of absence seizures (Kros et al., 2015). In contrast, and paralleling their optogenetic work, excitation (through application of the GABA_A_ antagonist gabazine) increased the firing frequency and regularity of deep cerebellar neurons and *inhibited* seizures. These findings, and previous work showing mixed results using open-loop electrical stimulation of the cerebellum in epilepsy (e.g., Cooper et al., 1973; Šramka et al., 1976; Van Buren et al., 1978; Levy and Auchterlonie, 1979; Wright et al., 1984; Davis and Emmonds, 1992; Chkhenkeli et al., 2004; Velasco et al., 2005), raise an important question: is the timing of intervention a critical factor? That is, if Kros et al. (2015) had used on-demand optogenetics to briefly and acutely inhibit deep cerebellar nucleus neurons, would they have worsened seizures (paralleling their pharmacological experiments; Kros et al., 2015), or instead inhibited seizures (paralleling the on-demand optogenetics work targeting the cerebellar cortex for TLE (Krook-Magnuson et al., 2014b), as well as the on-demand optogenetics work targeting the thalamus for thalamocortical epilepsy; Paz et al., 2013; Sorokin et al., 2017)? Fortunately, the temporal specificity achievable with optogenetic methods allows future examination of this important topic.

In these studies, the researchers not only used optogenetics to manipulate neuronal activity, they also used more traditional methods to record neuronal activity. Paralleling previous findings in a variety of seizure models (e.g., Fernandez-Guardiola et al., 1962; Julien and Laxer, 1974; Heath et al., 1980; Kandel and Buzsáki, 1993) as well as in some human patients (Niedermeyer and Uematsu, 1974), both groups found that activity in the cerebellum was modulated by seizures; both hippocampal temporal lobe seizures (Krook-Magnuson et al., 2014b) and thalamocortical absence seizures (Kros et al., 2015) were mirrored in the cerebellum. The ability to record spontaneous seizure activity in the cerebellum suggests that scientists and clinicians may need to reject the adage that the cerebellum “does not seize.” It may also warrant reconsideration of which brain regions are considered to be part of the core hippocampal and thalamocortical seizure networks. When targeting the cerebellum, are we targeting a region distinct from the seizure network which is merely able to inhibit seizures as an outsider? Or, is the cerebellum a core part of the seizure network? Optogenetics, in combination with other brain mapping tools, can help address these questions.

The cerebellum is certainly not the only brain region which has been examined as a potential site for inhibiting a variety of seizure types (Gale, 1992; Dybdal and Gale, 2000; Fisher et al., 2010; Connor et al., 2012; Valentín et al., 2013). One such brain region, which was recently examined using an optogenetic approach, is the superior colliculus—in particular, the deep/intermediate layers of the superior colliculus (Soper et al., 2016; Table 2). Optogenetic activation of neurons in the superior colliculus was able to inhibit seizures induced by: (i) systemic administration of pentylenetetrazole; (ii) focal bicuculline application to Area Tempestas in the piriform cortex; (iii) systemic administration of gamma butyrolactone; or (iv) acoustic stimulation in animals genetically prone to audiogenic seizures (Soper et al., 2016). These results support the ability of intervention targeted at the deep layers of the superior colliculus to be effective in a wide variety of seizure types. Notably, the superior colliculus is a downstream target of the cerebellum and in turn projects to areas that are also directly targeted by the cerebellum, suggesting that the two structures may be part of a unified seizure-controlling network (Yu and Krook-Magnuson, 2015; Zeidler and Krook-Magnuson, 2016). Optogenetics provides an important toolbox also for testing this possibility in future work.

### Optogenetic Cell-Type Selectivity for Local Circuit Dissection

The examples discussed above utilized the specificity achievable with on-demand optogenetic techniques to gain insight into the circuitry of epilepsy and seizures at a fairly macroscopic level—the importance of the dentate gyrus or thalamocortical interactions, and the relevance of other regions not classically associated with specific seizure networks. However, the cell-type specificity achievable with optogenetic techniques also allows probing of local circuit elements and the roles of different interneuron populations in healthy physiology and in neurological disorders such as epilepsy. In epilepsy research to date, these optogenetic tools have been most extensively used to investigate parvalbumin-expressing (PV) inhibitory neurons.

PV interneurons are a heterogeneous group of fast spiking inhibitory neurons, which together make up less than 5% of hippocampal neurons (Woodson et al., 1989; Freund and Buzsáki, 1996; Bezaire and Soltesz, 2013). While relatively few in number, PV cells are known to have a powerful impact in healthy circuits (Cobb et al., 1995; Freund and Buzsáki, 1996; Hajos and Paulsen, 2009; Sohal et al., 2009). Their role in seizures, however, has become somewhat controversial (Cammarota et al., 2013; Ledri et al., 2014; Maguire, 2015; Yi et al., 2015; Jiang et al., 2016). Using on-demand optogenetics with light delivery to the hippocampus and a model of chronic TLE, it was demonstrated that activation of PV cells selectively at the time of seizures, and early during seizures (before they progressed to having overtly behavioral manifestations), was able to significantly reduce the duration of electrographic seizures and reduce the likelihood that a seizure would progress to becoming a behavioral seizure (Krook-Magnuson et al., 2013). Seizures were significantly inhibited when light was delivered to the hippocampus ipsilateral to the presumed seizure focus or, somewhat surprisingly, to the contralateral hippocampus (Figure 5). This suggests that activation of PV cells can be an effective approach to inhibiting seizures. However, work done with evoked seizures in healthy tissue suggests that the role of PV cells may be more complicated than simply always providing seizure control. The effect of activating PV cells may be dependent on the timing of their activation (Ellender et al., 2014; Assaf and Schiller, 2016), the relative location of PV cell activation compared to the seizure focus (Sessolo et al., 2015), the type of seizure (Shiri et al., 2015, 2016; Lu et al., 2016) and/or the brain region targeted and expression levels of NKCC1/KCC2 (Wang et al., 2017). More work examining the role of PV neurons during spontaneous seizures *in vivo* in epileptic animals, as well as work parsing the role of different types of PV interneurons (as discussed more below) would help clarify these issues.

Optogenetics is a powerful tool for providing new insight into the role of populations of cells in neurological disorders. However, in the case of determining the role of PV interneurons during seizures, and to some extent interneurons more broadly, optogenetics has so far produced more questions than answers (Chiang et al., 2014; Ledri et al., 2014; Ladas et al., 2015; Yekhlef et al., 2015; Shiri et al., 2016; Khoshkhoo et al., 2017). Luckily, optogenetics also provides the opportunity to address these questions more thoroughly moving forward.

## Versatility

The optogenetic toolbox is filled with a wide variety of tools (Box 1; Figure 1) which can be used for different purposes to distinct experimental ends. Moreover, optogenetic techniques are typically relatively easy to implement in conjunction with more traditional methods, further increasing the versatility of use of optogenetic techniques. As discussed above, in the context of epilepsy research, optogenetic methods have been used to study networks in epilepsy through both seizure inhibition and seizure induction. To date, this has relied primarily on the widely used opsins HR and ChR2 (an overview of optogenetic methods currently used in *in vivo* epilepsy research is included in Table 2), and a full harnessing of the varied tools (Box 1) available is still to come, as discussed more in the “Further Development and Application” section below. Importantly, optogenetics has also been used in epilepsy research to monitor changes in networks in epilepsy and with interventions. To illustrate the versatility of optogenetics, we discuss here some of the ways optogenetics has been paired with other techniques to gain these insights. While our discussion focuses on epilepsy research, it is important to note that these methods are more broadly applicable (Table 1); for example, optogenetics has not only been used to track changes in neuronal circuits in epileptic tissue, but also following stroke (Lim et al., 2014; listed in Table 1).

Axonal sprouting is a key example of network reorganization that can occur in epilepsy. A classic example of this in TLE is axonal sprouting of dentate granule cells (i.e., mossy fiber sprouting; Laurberg and Zimmer, 1981; Tauck and Nadler, 1985; Sutula et al., 1989; Buckmaster and Dudek, 1997; Althaus et al., 2016). Inhibitory axonal sprouting can also occur (Mathern et al., 1995; Zhang et al., 2009; Peng et al., 2013; Soussi et al., 2015; Christenson Wick et al., 2017), and this is one area in which optogenetic techniques have been paired with more traditional methods of studying anatomical reorganization. Recently, Peng et al. (2013) selectively expressed the excitatory opsin ChR2 in SOM cells of CA1 by injecting a Cre-dependent virus (Figure 3Aii) into the dorsal hippocampus of healthy and epileptic SOM-Cre mice. Because the expression of the virus was limited to SOM cells in the stratum oriens of CA1 and because the opsin was fluorescently tagged, the authors were able to clearly view the axonal arbors of SOM cells in healthy and epileptic mice. Upon visualizing the fluorescently labeled axonal arbors, Peng et al. (2013) found that SOM cells displayed axonal sprouting in epileptic mice. In healthy mice, the axonal arbors of labeled SOM cells in CA1 stopped rigidly before the hippocampal fissure—not crossing into the dentate. However, in epileptic mice, labeled SOM cells in CA1 had axonal arbors that crossed the hippocampal fissure, invading the dentate gyrus. Using optogenetics to activate labeled cells, and whole cell patch clamp methods to record from dentate granule cells in hippocampal slices, Peng et al. (2013) confirmed that these sprouted axons made functional connections onto dentate granule cells in the epileptic mice. More recently, work by others has shown that some CA1 SOM cells have axons that cross into the dentate in non-epileptic tissue (Katona et al., 2017; Szabo et al., 2017), suggesting the possibility that the specific population of CA1 SOM cells targeted may be a critical factor. An additional experimental caveat to consider in using optogenetic approaches in epilepsy research is that seizures can alter the traditional expression profile of markers including somatostatin, which can be transiently expressed in principal cells following seizures (Drexel et al., 2012). As discussed in a later section, intersectional approaches (Figure 3iii) may help alleviate both of these experimental considerations in future work. Importantly, in the study by Peng et al. (2013), an optogenetic approach, in combination with more traditional methods, allowed the demonstration of a novel form of plasticity in chronically epileptic tissue. Additionally, invasion of inhibitory axons from CA1 SOM neurons into the dentate gyrus, including in epileptic tissue, highlights that not only may local inhibitory neurons be harnessed to reinforce the dentate “gate,” but also that inhibitory input from other regions invading the dentate may also be able to help support the gate. In this context, recent work from our laboratory looking at axonal sprouting of PV cells is of interest.

As noted above, it was previously demonstrated that optogenetic activation of PV cells contralateral to the presumed seizure focus significantly inhibited temporal lobe seizures (Krook-Magnuson et al., 2013). This was a somewhat surprising finding, as directly inhibiting granule cells in the contralateral hippocampus had no effect on the seizures (Krook-Magnuson et al., 2015a). There are several possibilities for why activating contralateral PV cells might produce seizure inhibition when direct inhibition of contralateral granule cells does not. One such potential explanation was that PV cells, despite being typically thought of as local interneurons, might project to the contralateral hippocampus (Goodman and Sloviter, 1992; Zappone and Sloviter, 2001), and provide “outsider” inhibition (somewhat akin to SOM cells invading the dentate from CA1). To test this, we injected the retrograde tracer Fluorogold into the hippocampus of control (non-epileptic) and kainate-injected (to induce TLE) mice (Christenson Wick et al., 2017). In control animals, we found only very sparse Fluorogold labeling in contralateral PV cells (~0.5% of contralateral PV cells displayed Fluorogold labeling). In animals injected with kainate 6 months earlier, however, we found a significant increase in Fluorogold-labeled contralateral PV neurons (Figure 5E). To further examine this issue, we used viral injection to achieve Cre-dependent expression of enhanced yellow fluorescent protein (eYFP) in PV cells, allowing visualization of their contralaterally projecting fibers (Christenson Wick et al., 2017). We found long-range sprouting of this commissural inhibitory connection 6 months post-kainate injection (Figure 5F). Specifically, dramatic increases (greater than 100× increase) in labeled fiber lengths were found in the dentate gyrus, further supporting the relevance of this area (and inhibition to it) in TLE. As these studies illustrate, the versatility and flexibility of optogenetic tools to work alongside currently existing well-established techniques greatly increases the power of optogenetics to allow relatively easy expansion on previous findings, including in the domain of circuit changes in epilepsy.

Circuit reorganization can also occur following treatment approaches for epilepsy, including cell transplantation. Here, too, optogenetics can be useful. Specifically, it has been shown that transplantation of interneurons or of progenitors of GABAergic neurons into adult epileptic mice can reduce seizures (Hunt et al., 2013; Cunningham et al., 2014; Henderson et al., 2014; Hunt and Baraban, 2015). When such transplants express opsins, and standard slice electrophysiology techniques are combined with optogenetics, it is possible to demonstrate that the transplanted cells integrate into the circuit and provide synaptic inhibition to endogenous excitatory cells (Avaliani et al., 2014; Cunningham et al., 2014; Henderson et al., 2014; Hsieh and Baraban, 2017). Again, it is worth noting that such research is certainly not limited to the field of epilepsy. For example, optogenetics to study the integration of grafted cells has been used to examine integration of stem cell-derived dopaminergic neurons in a model of Parkinson’s disease (Tønnesen et al., 2011; Table 1). Further illustrating the relative ease at which optogenetics can be combined with other approaches, opsin expression in transplanted neurons was recently used alongside functional magnetic resonance imaging (fMRI; when paired with optogenetics, referred to as ofMRI) to examine larger-scale integration of transplants (Weitz and Lee, 2016).

The technique of ofMRI has also been used in epilepsy research to examine seizure dynamics across brain areas (Duffy et al., 2015; Choy et al., 2017). While ofMRI research so far has focused on using optogenetics to induce seizures in the scanner, future research could use ofMRI to examine seizure networks with spontaneous (rather than evoked) seizures in epileptic animals and use optogenetics to inhibit those seizures to examine seizure suppressive networks. This would be useful, for example, in gaining a better understanding of the networks engaged by interventions targeting the superior colliculus or the cerebellum. Indeed, ofMRI has recently been applied to cerebellar optogenetic stimulation in healthy animals (Choe et al., 2018). As a side note, ofMRI work also provides an excellent example of the need to always have appropriate experimental controls, and specifically the possibility of off-target effects of light; a recent report suggests that light itself can alter blood flow (Rungta et al., 2017).

With appropriate consideration of activation spectra and other methodological considerations, optogenetics can also be combined with cellular level imaging techniques such as calcium imaging. Khoshkhoo et al. (2017) paired optogenetically induced cortical seizures with fiber photometry in order to monitor the activity of different populations of neurons contralateral to the hemisphere receiving the optogenetic activation. In particular, they examined PV, SOM, vasoactive intestinal peptide-expressing (VIP) neurons and excitatory neurons. They found that evoked seizures produced significant increases in calcium signals for all cell types studied, with inhibitory populations showing a calcium signal increase coinciding with electrographic seizure onset, and excitatory cells showing increases after several seconds of lag. For these optogenetically induced seizures, PV and SOM calcium signals remained high for the duration of the seizure events. In addition to optogenetically inducing seizures, Khoshkhoo et al. (2017) used optogenetics to then investigate the effect of inhibiting populations of neurons on evoked seizures and found mixed effects. An additional method to track the activity of neuronal populations during seizures, or potentially across the development of epilepsy itself, is “opto-tagging.” For this, optogenetics is paired with unit recordings, with responsiveness to light being used to determine the cell-type, supplementing putative identification based on waveforms or firing patterns. This use has led to the development of optetrodes, capable of delivering light while performing multichannel recordings (Anikeeva et al., 2011). Relevant to epilepsy research, opto-tagging allows *in vivo* monitoring of the activity of specific cell types with higher temporal resolution than, for instance, calcium imaging. In addition, recordings with optetrodes provide details on how the optogenetic manipulation is affecting the cells’ activity (i.e., is the cell entraining to the stimulus; is the cell rebounding after photostimulation; has the chloride potential shifted such that chloride channel opsins are depolarizing rather than hyperpolarizing, etc.).

The examples discussed above demonstrate how optogenetics can be used in flexible ways and in combination with different technologies to illustrate its versatility. However, given the large diversity of optogenetic tools currently available (Box 1), and the continued development of new tools, the use of optogenetics in epilepsy research to date represents only a small fraction of what is possible. Continued development, alongside the use of the more varied tools, will allow epilepsy research to fully harness the versatility of optogenetic approaches. These issues are considered below.

## Further Development and Application

There are several areas in which optogenetic techniques are being further developed. These include: (1) the light-sensitive proteins themselves and what they couple to; (2) methods for delivering light; and (3) methods for improving selectivity of expression (see also Box 2). Box 1 discusses classical opsins as well as more recently developed tools. Methods for delivering light, beyond traditional optical fibers, include μLEDs (Kim et al., 2013), transparent microprobe arrays (Lee et al., 2015), and wireless devices (for reviews, see Warden et al., 2014; Gutruf and Rogers, 2018). Light delivery approaches also include methods which take a fundamentally different approach, such as luminopsins (Tung et al., 2015) or upconversion nanoparticles (Chen et al., 2018; both briefly discussed in Box 1). A combination of fully harnessing available tools and development of new tools will allow optogenetics to make a significant impact on epilepsy research moving forward. Here, we discuss some ways in which newly developed, and not-yet developed, tools could be applied to epilepsy research to further the field, particularly focusing on improved cell-type specific targeting.

Increased cell-type specific targeting is certain to have a major impact on epilepsy research. For example, relatively little work has been done examining SOM-expressing inhibitory neurons in epileptic tissue (the work by Peng et al., 2013 discussed above is a notable exception). One major reason for this dearth of research is that GABAergic SOM neurons are difficult to selectively label and manipulate in animal models of epilepsy that display transient SOM expression in pyramidal cells (Hashimoto and Obata, 1991; Drexel et al., 2012). Use of Cre-based systems (Figure 3), in which opsin expression is Cre-dependent and Cre-expression is placed under the SOM promoter is problematic in these circumstances, as even transient expression of SOM (and thus Cre) in pyramidal cells is sufficient to irreversibly induce expression of the opsin in these excitatory cells. Therefore, maintenance of selective expression in inhibitory SOM neurons is compromised.

However, the increasing availability of Cre- and Flp-recombinase expressing mouse lines and systems (Taniguchi et al., 2011; Madisen et al., 2012, 2015; Tang et al., 2015; He et al., 2016) and recent advances in intersectional approaches for the restricted expression of opsins (Figure 3iii; Fenno et al., 2014) finally allow for more selective targeting and manipulation of cell populations. Importantly, these intersectional tools can aid in maintaining consistent selective expression in epileptic tissue. For instance, by crossing SOM-Cre mice with Dlx5/6-Flp mice, one can produce mice in which inhibitory SOM neurons express both Cre- and Flp-recombinase. Then, to selectively target and manipulate these neurons in a given brain region, one could inject an INTRSECT virus expressing the opsin of choice in a manner dependent on both Cre- and Flp-mediated recombination (Con/Fon; Figure 3iii). In this situation, transient expression of SOM in non-GABAergic populations would not compromise specificity of expression, as non-GABAergic populations would not express Flp and therefore not express the opsin. Therefore, through application of already available tools, the epilepsy field can harness optogenetic techniques to study populations of cells previously difficult to selectively target.

While the example above focuses on SOM cells in epileptic tissue, such intersectional approaches can also be used to examine other GABAergic populations that have been traditionally difficult to study, such as cholecystokinin-expressing (CCK) or neuronal nitric oxide synthase-expressing (nNOS) interneurons. While CCK and nNOS are both used as markers of certain populations of interneurons (Freund and Buzsáki, 1996; Armstrong et al., 2012), both are also natively expressed in excitatory principal cells (Burette et al., 2002; Lee and Soltesz, 2011). Therefore, targeting these cell populations with a traditional Cre-based system is insufficient. This has been a major problem for the field, but one which is now relatively straightforward to overcome; all the necessary tools are available to optogenetically excite populations of interneurons defined by their expression of molecular markers such as CCK or nNOS.

However, an additional hurdle for the field, alluded to earlier, is that neuronal markers such as SOM, PV, CCK or nNOS are fairly broad markers, with several distinct cell-types encompassed by each marker. For example, PV is expressed in hippocampal fast-spiking perisomatic targeting basket cells, in some dendritically targeting bistratified and oriens-lacunosum moleculare (O-LM) cells, and in axon-initial-segment targeting chandelier (axo-axonic) cells (Freund and Buzsáki, 1996; Bezaire and Soltesz, 2013). Each of these neuronal populations target different segments of the principal cell and may even have diametrically opposing effects on the postsynaptic cell. For instance, axo-axonic cells may actually increase the firing of the postsynaptic cell (Szabadics et al., 2006). Moving forward, it will be critically important for the field to gain better selectivity—to stop targeting “PV cells,” and start targeting more specifically defined cell populations. This may also help clarify the different outcomes seen when targeting broadly defined PV cells in epilepsy research (discussed above). Here too, intersectional approaches can be of great value. For example, in addition to expressing PV, bistratified and O-LM cells can express SOM (Freund and Buzsáki, 1996). Therefore, an intersectional approach, using PV-Flp or SOM-Flp mice in combination with SOM-Cre or PV-Cre mice, respectively, and INTRSECT viruses (Con/Fon, Coff/Fon, Con/Foff, Coff/Foff) can limit expression to subpopulations of PV cells (Fenno et al., 2014). Despite the progress this represents, selective targeting of just PV basket cells, for example, is not yet achievable, and will require the continued development of new tools. The need for continued development of new tools includes tools and insight not directly associated with optogenetics. For example, new mouse lines based on cell-type specific expression profiles gleamed through single-cell transcriptomics can provide important new avenues to specific opsin expression: a recent study identified neuron-derived neurotropic factor (Ndnf) expression in cortical neurogliaform cells, and then produced a Cre-line to allow targeting of this neuronal population (Tasic et al., 2016). While not directly an optogenetic development, this insight and new tool (the Ndnf-Cre line) allows the expansion of optogenetic methods. The same study which identified Ndnf-expression in cortical neurogliaform cells suggested that PV cells expressing Cpne5 (copine 5, a calcium-dependent protein) may correspond to axo-axonic cells (Tasic et al., 2016), which could provide a genetic handle for separating different classes of PV interneurons in the future.

In addition to gene-expression profiles, cell populations can also be segregated by projection targets. While the discussion above has focused on the use of intersectional approaches to improve cell-type specificity through the use of Flp and Cre mouse lines, intersectional approaches can be used in combination with other methods, including to selectively express opsins in cell populations defined by their projection targets. This development increases optogenetics’ power for exploring circuit-level interactions between brain regions in health and disease. For example, Cre and/or Flp expression can be achieved through retrograde expression systems, such as CAV2 (Junyent and Kremer, 2015), modified rabies (Wall et al., 2010; Chatterjee et al., 2018), or AAV2-retro (Tervo et al., 2016; Box 2, Figure 6). In this manner, dopaminergic neurons in the VTA that project to the nucleus accumbens (NAc) were selectively targeted in the original INTRSECT publication (Fenno et al., 2014). One potential application for an optogenetic examination of projection-defined neuronal populations in epilepsy is to explore the distinct contributions of deep vs. superficial CA1 pyramidal cells to epileptic neural activity, including seizure spread or generalization of spikes (Sheybani et al., 2018). Deep vs. superficial CA1 pyramidal cells project to distinct areas (Slomianka et al., 2011), interact with distinct local circuit elements (Krook-Magnuson et al., 2012; Lee et al., 2014), and display different neuromodulatory pathways, including via cannabinoid type-1 receptors (Maroso et al., 2016). For a review on CA1 pyramidal cell diversity, see reference (Soltesz and Losonczy, 2018). As deep and superficial pyramidal cells have distinct projection targets, it is possible to use a retrograde expression system to selectively label and manipulate just one population of pyramidal cells. This would not necessarily require an intersectional approach, but by combining retrograde-expression with an additional restriction (e.g., promoter-based Cre expression), opsin expression could be further limited. Such an approach would be useful, for example, in studying GABAergic projection neurons in situations where the target region is also targeted by excitatory principal neurons, such as entorhinal cortex inputs to the hippocampus (Melzer et al., 2012; Basu et al., 2016).

In addition to increased opsin delivery tools, increases in the availability of diverse light-sensitive proteins make it possible to combine two distinct optogenetic approaches within the same animal. For instance, the increasing availability of good red-shifted and blue-light activated opsins (Figure 2, Box 1) allow for simultaneous/dual-opto experiments in which two cell populations can be independently targeted and then independently manipulated. Building on the discussion of different targeting approaches, this dual-opto approach could be applied to epilepsy research in several ways. For instance, one could retrogradely target, and then manipulate, deep and superficial CA1 pyramidal cells *independently, in the same animal*. To do this, one could inject a CAV (or other retrograde) virus carrying a blue-light activated opsin (e.g., Chronos) into the NAc (to target deep pyramidal cells; McGeorge and Faull, 1989) and inject a CAV virus carrying a red-shifted opsin (such as Chrimson) into the medial temporal cortex (mTC; to target superficial pyramidal cells; Slomianka et al., 2011; Figure 6). Because the activation spectra of these two opsins are sufficiently distinct, one could activate one opsin without activating the other by using different wavelengths of light. With such an approach, one could examine if a given postsynaptic cell within CA1 was targeted by both populations of pyramidal cells, or selectively by one population or the other, and how synaptic reorganization associated with epilepsy might disrupt deep and superficial pyramidal cell wiring. To date INTRSECT has only been published for fluorescent proteins and ChR2 (Fenno et al., 2014). With further development of INTRSECT vectors to include additional opsins, INTRSECT approaches could be applied in dual-opto situations or in cases benefiting from inhibitory opsins.

The examples provided here are merely examples—the opportunities for combinatorial approaches that current and developing optogenetic tools provide are nearly endless. Clearly, optogenetic approaches are experimentally powerful, and will continue to be so moving forward.

While we have focused on the possibility for expansion of cell-type specific targeting of optogenetic methods in epilepsy research, and while the majority of optogenetic-aided epilepsy research (Table 2), and to some extent optogenetic-aided study of neurological disorders more broadly (Table 1), has been focused at the circuit level, use of optogenetics for molecular-level research is becoming increasingly possible, and deserves a brief discussion here. Indeed, several optogenetic tools are already available that would be very useful for studying neurological disorders, including epilepsy, from a molecular vantage (Box 1). These tools include light-activated G-protein coupled receptors (Opto-XRs; Airan et al., 2009), as well as a more diverse and ever-expanding tool box of specialized light-sensitive proteins.

One line of optogenetic tools which may be particularly useful for investigations at a more molecular level are systems in which light induces association or dissociation of proteins (Figure 1F, Box 1). There are a variety of these, including OptoSOS and the Phy-PIF system (Toettcher et al., 2013), LITEs and the CRY2-CIB1 system (Konermann et al., 2013), mutated Dronpa-based caging (Zhou et al., 2012), VP-EL222 and light-oxygen-voltage (LOV) domains (Motta-Mena et al., 2014), LOVTRAP (Wang et al., 2016), and photoactivatable Cre (Kawano et al., 2016). A recently developed system, PhoCl, is somewhat similar but rests on light-induced cleavage of a protein, making for a direct irreversible alteration by light (Zhang et al., 2017). The availability and advances to tools using light-induced alterations in protein-protein associations (or photocleavage of proteins) increases the possibilities for how optogenetics can be used to study the nervous system more broadly, by directly influencing the activity of intracellular proteins. As with other optogenetic approaches, these approaches can be used with the temporal and spatial specificity afforded through the use of light, and the cell-type specificity achievable through genetic encoding. Another use for systems in which light alters protein-protein interactions is to directly alter gene expression (e.g., LITEs (Konermann et al., 2013) and VP-EL222 (Motta-Mena et al., 2014)) or DNA itself (e.g., through a photoactivatable Cre system (Kawano et al., 2016)). Such light-based approaches may be able to achieve greater temporal specificity and reduced complications compared to more traditional methods like the Tetracycline/tamoxifen systems (Saunders, 2011). These tools are currently available, but not yet adopted in epilepsy research.

Additionally, the continued development and sharing of optogenetic tools, including optogenetic pharmacological tools, increases the potential for optogenetics as a strategy in cases where pharmacological tools may be lacking. In optogenetic pharmacology, a receptor (or subunit of a receptor) of interest is modified (e.g., through a single cysteine substitution) to allow a chemical photoswitch to (selectively and irreversibly) tether, thus combining genetic and chemical approaches. The photoswitches can be engineered to act as either an agonist or antagonist of the receptor in the absence or presence of a given wavelength of light. Previously, optogenetic pharmacology was available for various potassium channels (e.g., “SPARK” (Banghart et al., 2004)), glutamate receptors (e.g., “LiGluR” (Volgraf et al., 2006)) and acetylcholine receptors (e.g., “MAACh” (Tochitsky et al., 2012)). An optogenetic pharmacology approach was also recently developed for GABA_A_ receptors (Lin et al., 2015). While the use of optogenetic pharmacology methods are arguably less straightforward than some other approaches, it offers the unique possibility of manipulating *a select type of receptor* in a particular cell type, in a particular brain region, at a particular time, and could be useful in the study of subunit specific contributions to epilepsy.

In addition to these optogenetic pharmacological tools, there have also been developments in other light-sensitive tools that may be useful where traditional pharmacological tools are currently lacking. Recently, Zhang et al. (2017) developed a photoactivatable version of the large-pore channel pannexin-1, which they termed Opto-Panx1. This tool could be directly relevant to research investigating pannexins or ATP release (as Opto-Panx1 activation induces ATP release) in health or disease states. Relevant to epilepsy research, ATP release is known to propagate glial calcium waves (Cotrina et al., 1998), which have been suggested to play a role in seizures (Gómez-Gonzalo et al., 2010). Also of note, glial calcium waves are blocked by some conventional anti-epileptic drugs (Tian et al., 2005). The potential impact of photocleavable proteins extends beyond pannexins and ATP, however. For an example, consider connexins. Connexins and the role of gap junctions in epilepsy is notoriously difficult to study owing to the lack of drugs that specifically and cleanly target connexins. While no Opto-connexin yet exists, one can easily imagine the potential uses for such a tool, and this is therefore an example of how continued tool development can aid future epilepsy research.

In addition to tools specifically designed to manipulate cellular processes at a molecular level, the currently widely available tools typically used for circuit-level investigations have been cleverly used in some instances for the study of more molecular phenomena. For example, in a stroke study by Beppu et al. (2014; listed in Table 1), the authors used ChR2(C128S) to induce inward flow of H+, and the inhibitory opsin ArchT to pump H+ out of the cell, in order to manipulate pH in glial cells. By optogenetically manipulating the acidity of glia in this manner, the authors demonstrated an impact on neurodegeneration at the site of ischemia. This is additionally an example of how considering how optogenetics has been used outside of the field of epilepsy (Table 1) could inspire future uses of optogenetics in epilepsy research.

The optogenetic tools available, both in terms of the opsins themselves and methods to achieve selective opsin expression, are rapidly expanding. As with any quickly growing field (or arguably any method), and discussed more in Box 1, optogenetics has limitations and considerations that must be acknowledged and controlled for in experimental design, including controlling for non-specific effects of light (including heat or sensory stimulation); expression of Cre, Flp, fluorescent proteins, or opsins themselves; potential effects of viral-based delivery; and “off-target” effects, including changes in ionic concentrations. Moreover, development of new tools is only one step in the process; the application of the tools to epilepsy research will depend in large part on the continued broad availability of the tools, and thus relies on tool developers making the conscious decision to make their tools widely available. With the specificity achievable with optogenetics, the versatility of optogenetic approaches, and the continued development (and sharing) of new tools, optogenetics will have a lasting impact on epilepsy research.

## Conclusion

Optogenetics is a quickly growing field that provides researchers with the ability to manipulate neurons with unprecedented specificity. It is also a versatile tool, easily used in combination with other techniques to answer diverse experimental questions. Continued development and sharing of new methods, including for selective opsin expression, ensure the further expansion of optogenetics’ utility in epilepsy research.

## Author Contributions

ZCW and EK-M drafted the manuscript.

## Conflict of Interest Statement

The authors declare that the research was conducted in the absence of any commercial or financial relationships that could be construed as a potential conflict of interest.

## Footnotes

^1^“Vectors that produce retrograde labeling” should not be confused with the term “retrovirus,” which instead refers to a family of single-stranded RNA viruses, including HIV and lentiviruses.
